# Non‐Noble Metal Nanocatalysts for Hydrogen Evolution

**DOI:** 10.1002/advs.76286

**Published:** 2026-07-17

**Authors:** Lina Jaya Diguna, Gede Herry Arum Wijaya, Md Abdul Kuddus Sheikh, Tobias Haposan, Fidelis Stefanus Hubertson Simanjuntak, Arramel Arramel, Muhammad Danang Birowosuto, Konstantin Sergeevich Novoselov

**Affiliations:** ^1^ Department of Renewable Energy Engineering Universitas Prasetiya Mulya Tangerang Indonesia; ^2^ Łukasiewicz Research Network‐PORT Polish Center for Technology Development Wrocław Poland; ^3^ Center of Excellence Applied Physics and Chemistry Nano Center Indonesia Tangerang Indonesia; ^4^ Institute For Functional Intelligent Materials National University of Singapore Singapore Singapore

**Keywords:** atomic doping, defect engineering, hydrogen evolution reaction, nanocatalysts, non‐noble metal

## Abstract

Hydrogen is a promising clean energy vector with a pivotal role toward a sustainable and renewable energy future. The latest advancement in hydrogen evolution reaction is mainly governed through photocatalysis, thermocatalytic steam reforming, and electrocatalysis. Yet the reliance on noble metal catalysts, particularly platinum, remains a key challenge due to their high cost and scarcity. Recent advancements in material science and nanotechnology introduce non‐noble metal‐based nanocatalysts (transition metal phosphides, sulfides, carbides, and nitrides), offering advantages of earth abundance, cost‐effectiveness, and tunable properties. This review explores the current progress and future prospects of non‐noble metal‐based nanocatalysts for hydrogen production. Advanced characterization tools, such as ultrafast pump‐probe spectroscopy and scanning electrochemical microscopy, provide unprecedented insights into charge carrier dynamics and active site distributions, linking nanoscale structures to catalytic performance. Beyond electrocatalysis, the integration of photocatalytic and thermocatalytic systems with renewable feedstocks is highlighted as a pathway for broader sustainability goals. By addressing challenges in catalyst design, synthesis, and characterization, this work underscores the potential of non‐noble metal‐based nanocatalysts to rival noble metals, paving the way for transformative advancements in hydrogen production and energy technologies. This comprehensive analysis aims to bridge knowledge gaps and inspire innovative strategies for a sustainable clean hydrogen economy.

## Graphical abstract

Recent overviews and latest developments of diverse classes of non‐noble catalysts for application toward high‐performance photocatalysis, thermocatalytic steam reforming, and electrocatalysis. Toward sustainability objectives, several advanced characterization tools in combination with the latest breakthroughs in machine learning for material discovery, here, we outline innovative approaches for a sustainable clean hydrogen economy.

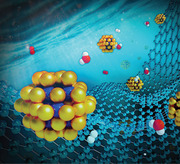

## 1 Introduction

Over the past few decades, nanocatalysts have emerged as indispensable components in advancing catalytic processes, extending far beyond conventional industrial applications. Their unique physicochemical properties, such as high surface area, tunable electronic characteristics, and robust catalytic activity, position them as key materials for addressing global challenges in energy and environmental sustainability. However, rationally designing nanocatalysts that meet the stringent requirements for enhanced activity, selectivity, and stability remains an immense challenge [1, 2, 3, 4]. These challenges are particularly pronounced in the search for chemically active agents capable of driving sustainable hydrogen (H_2_) production, a clean and versatile energy carrier.

In the past few years, enormous interest in catalysts has led to diverse applications spanning from water‐splitting processes [5, 6, 7, 8] toward sustainable technologies [9]. The hydrogen evolution reaction (HER) has garnered significant attention as a vital pathway for achieving efficient H_2_ production. Noble metals like platinum are widely recognized as benchmark catalysts for HER due to their near‐zero overpotential and outstanding catalytic efficiency [10, 11, 12, 13]. However, their high cost and limited availability have catalyzed research into alternative non‐noble metal‐based nanocatalysts, including transition metal phosphides (e.g., Ni_2_P), sulfides (e.g., MoS_2_), carbides (e.g., WC), and nitrides (e.g., VN) [14, 15, 16, 17, 18, 19]. These materials offer the advantages of earth abundance, cost‐effectiveness, and tunable properties, but require further optimization to match the performance of noble metals.

Recent advancements in nanomaterial synthesis have led to the development of innovative strategies to enhance catalytic performance. For example, the use of heteroatom doping has improved the conductivity and active site density of MoS_2_ nanosheets, enabling enhanced HER performance in acidic and alkaline media [16, 20, 21, 22, 23]. Similarly, engineering heterostructures has been shown to promote electron transfer and boost catalytic activity [24]. Advanced synthesis frameworks have further expanded the accessible phase of transition metal nanocatalysts, demonstrating how controlled precursor chemistry and intercalation‐based exfoliation could produce well‐defined nanosheets with exceptional reproducibility [25]. These strategies demonstrate the potential of nanoscale engineering in overcoming intrinsic limitations of non‐noble metal catalysts. This review aims to provide an in‐depth exploration of the progress and potential of non‐noble metal nanocatalysts in H_2_ production. Here we provide a rigorous framework for both non‐specialists and researchers working across related disciplines, establishing a coherent perspective on high‐performance renewable energy systems for H_2_ production. We present a critical and comprehensive synthesis of the principal catalytic pathways thermocatalysis, photocatalysis, and electrocatalysis which differ fundamentally in their energy inputs and feedstocks but are unified by their reliance on catalyst properties to mediate and accelerate chemical transformations. Rather than reiterating the fundamental mechanisms of hydrogen energy production comprehensively reviewed in prior works [26, 27, 28, 29, 30, 31, 32, 33, 34, 35, 36, 37], we focus on the physicochemical principles governing hydrogen production and highlight key advancements in nanocatalyst development. The subsequent sections address critical topics, including the chemical and physical processes of HER, catalytic performance parameters, and the mechanistic roles of photocatalysis, thermocatalytic, steam reforming, and electrocatalysis. For instance, the Sabatier principle and volcano plots are the two parameters commonly discussed to elucidate the theoretical basis for optimizing catalytic activity.

**FIGURE 1:**
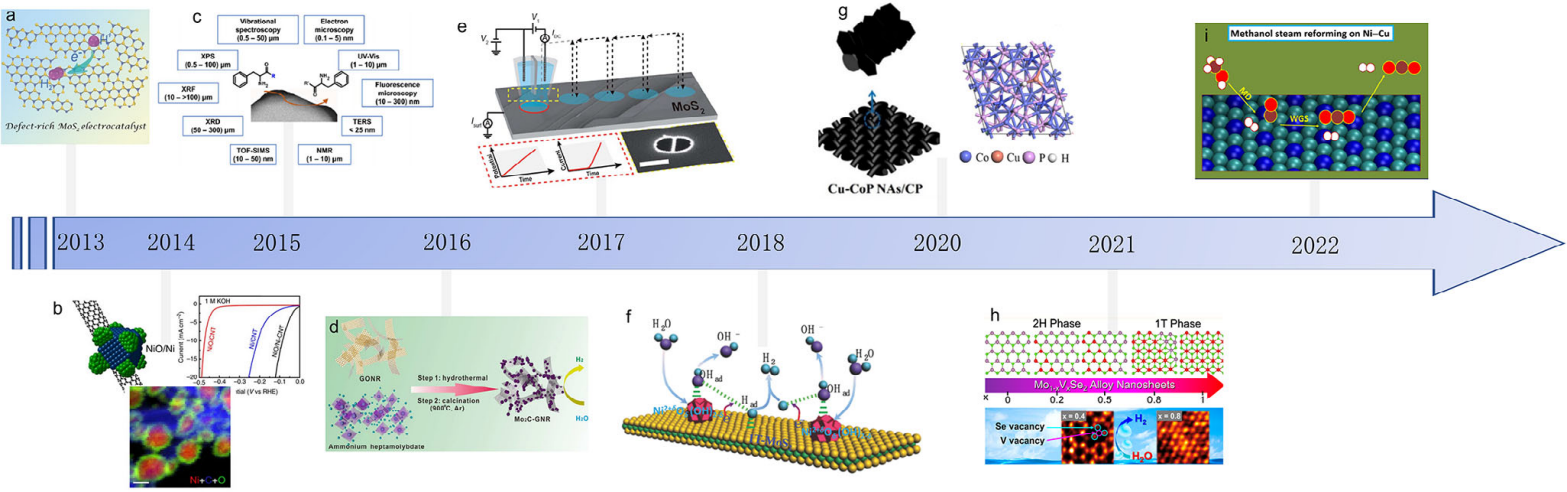
Chronology of breakthroughs in non‐noble metal nanocatalysts. (a) Defect‐rich induced HER production formed on carbon nanotubes, HER activity to enhance electrical conductivity using ultrathin MoS_2._ Reproduced with permission [41]. Copyright 2013, Wiley‐VCH GmbH. (b) Nanoscale nickel oxide/nickel heterostructures with high catalytic activity. Reproduced with permission [42]. Copyright 2014, Nature Portfolio. (c) Multitude of characterisation tools used to monitor HER. Reproduced with permission [43]. Copyright 2015, The Royal Society of Chemistry. (d) HER demonstration of molybdenum carbide (Mo_2_C) nanoparticles anchored on graphene nanoribbons (GNRs) operated in all‐pH conditions. Reproduced with permission [44]. Copyright 2016, American Chemical Society. (e) A distinct HER activity between the basal and edge plane of MoS_2_ using voltammetric scanning electrochemical cell microscopy (SECCM) technique. Reproduced with permission [45]. Copyright 2017, The Royal Society of Chemistry. (f) Hybridization of 0D nickel hydr(oxy)oxide nanoparticles with 2D metallic MoS_2_ nanosheets. Reproduced with permission [46]. Copyright 2018, Wiley‐VCH GmbH. (g) The effect of copper doping of CoP in facilitating the H* adsorption and desorption. Reproduced with permission [47]. Copyright 2020, Elsevier B.V. (h) Phase modulation of Mo_1−x_V_x_Se_2_ alloy nanosheets in the electrocatalytic HER. Reproduced with permission [48]. Copyright 2021, American Chemical Society. (i) A theoretical work of methanol steam reaction (MSR) mechanism on Ni−Cu surfaces. Reproduced with permission [49]. Copyright 2022, American Chemical Society.

To date, the current progress of state‐of‐the‐art characterization techniques such as ultrafast pump‐probe spectroscopy have provided valuable insights into charge carrier dynamics, while scanning electrochemical microscopy (SECM) has enabled real‐space visualization of active sites at the nanoscale [11, 26, 38, 39, 40]. These nanoscopic tools have significantly advanced our understanding on the structure‐activity relationships of nanocatalysts to unravel HER with unprecedented resolution. Finally, we examine the emerging role of low‐dimensional materials (LDMs), such as transition metal dichalcogenides (TMDs, e.g., MoSe_2_) and heterostructure catalysts, in enhancing HER performance. Emphasis is placed on scalable synthesis, interfacial engineering, and electronic structure optimization as critical pathways for further progress. By presenting a comprehensive analysis of current developments and offering a forward‐looking perspective, this work aims to contribute to the rational design of high‐performance, non‐noble metal‐based nanocatalysts, paving the way toward a sustainable clean hydrogen economy. To stimulate the review, we present a timeline comprising of several pioneering HER works related to the early development of non‐noble metal‐based nanocatalysts is depicted in Figure 1a–i.

## 2 Catalytic Mechanisms of Green H_2_ Production

H_2_ production is fundamentally a process of breaking chemical bonds, specifically C─H in hydrocarbons/biomass or O─H bonds in as water, to release hydrogen atoms and reorganize them into stable H_2_ molecules. Different pathways such as thermochemical, photocatalytic, and electrocatalytic methods depend on how energy is supplied and how electrons and protons are manipulated. Thermochemical methods use heat to shift reaction equilibria and overcome activation barriers, photocatalytic systems use light to excite electrons in semiconductors, and electrocatalytic methods use electricity to drive redox reactions. The optimization of H_2_ production requires catalysts with a Gibbs free energy of hydrogen adsorption close to zero, ensuring that hydrogen intermediate binding is neither too strong (blocking sites) nor too weak (preventing reduction). In all cases, the process depends on efficient charge transfer, catalytic lowering of activation energy, and overcoming the free energy requirements defined by Gibbs free energy to ultimately produce H_2_ gas. In addition to the catalyst type the catalytic loading, the specific amount of catalyst employed to a reactor or electrode surface to facilitate the chemical or electrochemical reaction in producing a specific quantity of H_2_, is also critical that directly influences the reaction rate, efficiency, and commercial viability of the H_2_ production system.

### 2.1 Thermocatalytic H_2_ Production

While H_2_ can be derived from various sources including water electrolysis and biomass, the majority of global production currently relies on thermochemical processes utilizing fossil fuel feedstocks like natural gas and coal due to their accessibility and established infrastructure. The strategic position of thermocatalytic mechanisms is therefore to refine these traditional pathways and adapt them for renewable feedstocks to balance immediate energy demands with long‐term decarbonization goals [50]. The role of catalysts in these thermochemical conversions is fundamental, as they are required to improve reactant conversion and selectivity in reactions that are often limited by thermodynamic equilibrium. Thermocatalytic conversion, particularly when applied to biomass or integrated with carbon capture and storage (CCS), offers a pathway toward “green” or low‐carbon hydrogen production that can utilize existing industrial frameworks. These mechanisms provide the necessary chemical pathways to break down complex hydrocarbons and alcohols into H_2_‐rich gas streams. By optimizing catalyst performance and reactor design, it is possible to enhance yields and reduce the energy penalties associated with high‐temperature operations [51]. Understanding these thermocatalytic principles is essential before examining specific applications, such as the widely used steam methane reforming and the emerging field of steam ethanol reforming that will be discussed in the subsequent sections.

#### 2.1.1 Steam Methane Reforming

Steam methane reforming (SMR) is the most common industrial process used for almost 50% of the world's hydrogen production [52]. This process involves reacting methane with steam to produce hydrogen, carbon monoxide, and a small amount of carbon dioxide. SMR constitutes the predominant industrial route for hydrogen production, serving as a cornerstone for synthesizing ammonia, methanol, and other chemical derivatives. This process, characterized by its simplicity and economic viability, has established itself as the standard within the chemical industry. The reaction process of SMR in producing hydrogen can be seen in equations (1) and (2).

(1) $$CH_{4}+H_{2}O↔\mathrm{CO}+3H_{2}$$

(2) $$\mathrm{CO}+H_{2}O↔CO_{2}+H_{2}$$

**FIGURE 2:**
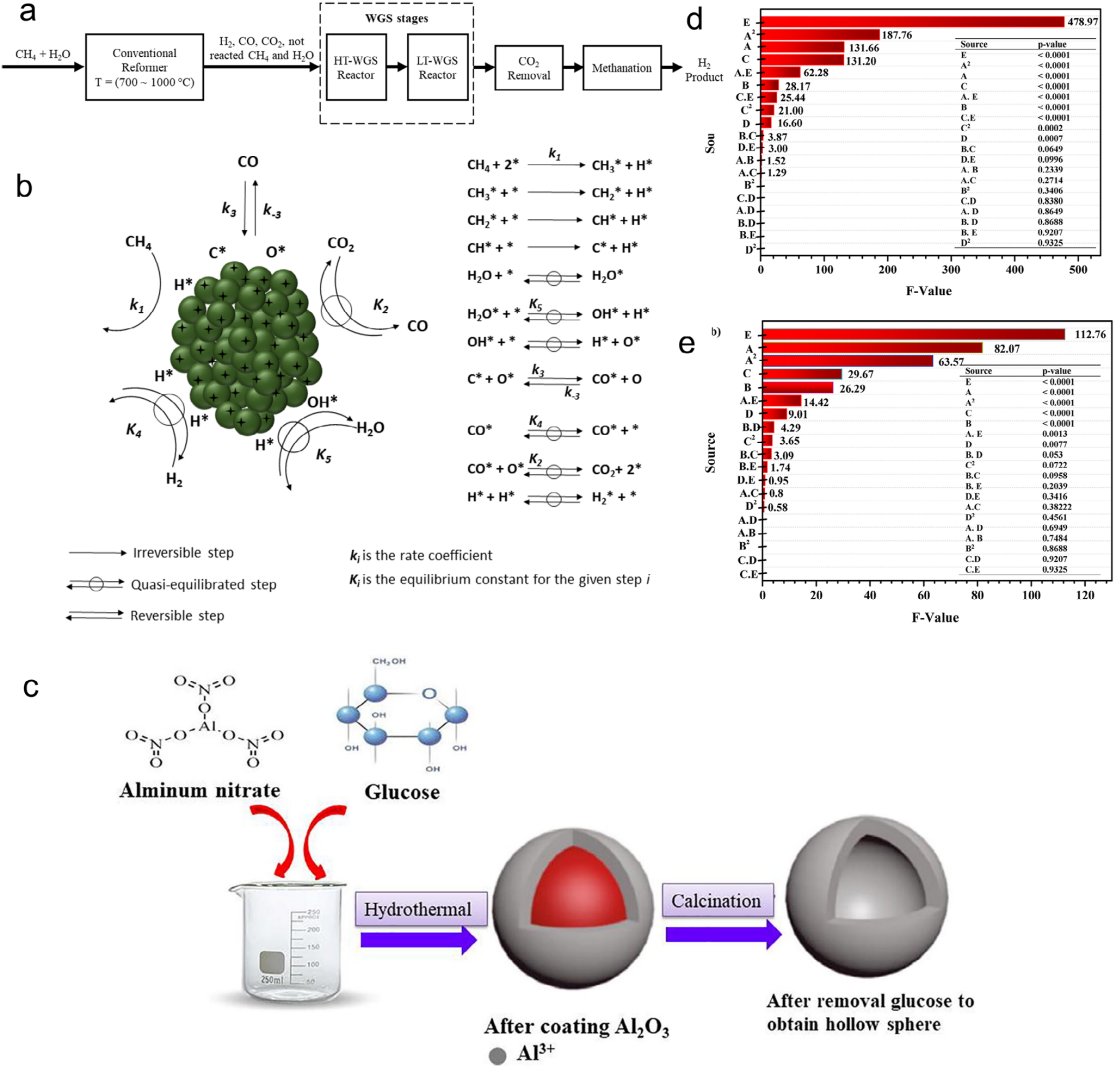
Methane steam reforming and catalyst performance for hydrogen production. (a) Scheme of methane steam reforming process for hydrogen production. Reproduced with permission [54]. Copyright 2004, Elsevier B.V. (b) Illustration of elementary steps for CH_4_ reforming and WGS reactions on Ni‐based catalysts. Reproduced with permission [55]. Copyright 2023, Elsevier B.V. (c) Synthesis scheme of Al_2_O_3_ hollow microspheres. Reproduced with permission [56]. Copyright 2022, Elsevier B.V. The effect of each parameter on the response of (d) CH_4_ conversion and (e) H_2_ yield; graded using the ANOVA result. Reproduced with permission [57]. Copyright 2023, Elsevier B.V.

The SMR process is a complex chemical reaction used to produce hydrogen gas. While it is the primary method for hydrogen production, it faces challenges like carbon deposition, which can deactivate the catalyst. The water‐gas shift (WGS) reaction is a secondary reaction that helps increase hydrogen yield by further converting CO and generating H_2_. Key factors influencing the process include pressure, temperature, water‐to‐carbon ratio, and space velocity [53]. A schematic of the SMR process in producing hydrogen can be seen in Figure 2a.

Nickel‐based catalysts, one of the most well‐known for their low cost and high catalytic activity, remain the predominant choice for the SMR process. Despite their advantages, challenges such as carbon formation (coking) and sintering of nickel particles happen in the low temperature condition. To address these issues, extensive research has focused on developing innovative nickel catalyst systems, including promoter‐modified, solid‐solution, novel‐supported, and self‐supported variants of Ni catalysts [58]. The proposed elementary steps for CH_4_ reforming and WGS reactions on Ni‐based catalysts are illustrated in Figure 2b. The symbols C* and H* denote the chemisorption of carbon and hydrogen species, respectively. Recent studies have demonstrated substantial advancements in the development of nickel‐based catalysts for SMR. As can be seen in Table 1, these catalysts exhibit notably enhanced performance in recent years, particularly in terms of methane conversion and hydrogen yield value.

**TABLE 1:** Selected papers published on Ni‐based catalysts for SMRs from 2017–2024.

Catalyst	Temp. (°C)	Conversion (%)	H_2_ yield (%)	H_2_ selectivity (%)	Gas hour space velocity (GHSV)	Year	Ref.
Ni/MgAl_2_O_4_	600	51.5	—	—	—	2017	[59]
Ni‐Pd‐0.1CNT	850	85.3	—	—	—	2018	[60]
Ni‐MgAl (CO‐IM) Ni‐MgAl (IM) Ni‐MgAl (CO)	850	75.5 60 38.5	65 51 41.5	—	60 L g^−1^ h^−1^	2019	[61]
Co‐Ni/CeO_2_	700	76.1	44.5	58.5	3 000 h^−1^	2020	[62]
Ni/La_0.7_Mg_0.3_AlO_3‐d_ (perovskite)	650	72.08	61.18	78.12	—	2021	[63]
Ni_2_‐Co_1_/H‐Al	700	90.72	93.82	—	37 500	2022	[64]
19.2Ni‐3.0Y/Al 16.2Ni‐2.7Y/HS‐Al	700	87 92	94 97	—	—	2023	[57]
Ni/Nb–Al_2_O_3_ (H)	550	55.74	41	—	14 400 mL/(g∙ h)	2023	[65]
0.17Co–Ni‐1.26Ce@H–Al	667	96.20	96.89	—	37 000 mL g^−1^h^−1^	2023	[66]
Ni_3_Cu_1_/Al_2_O_3_	800	87	62	—	15 000 h^−1^	2024	[67]
Ni‐B_0.5_/MgAl_2_O_4_	700	90.2	85	—	18 000 mL·(gh)^−1^	2024	[68]
Ni_CeO_2__Al_2_O_3_/OB‐SiC	720	99	85	—	10 h^−1^	2024	[69]
1Ni_4.5_Co_4_._5_CaO	600	∼100	99.6	—	—	2024	[70]

Research on nickel‐based catalysts for SMR is increasing with various strategies such as searching for suitable support catalysts, preparation methods, and combining two or more types of metals. Chaici et al. developed a novel supportless Ni‐Pd‐0.1CNT foamy nanocatalyst using electroless deposition on polyurethane support. The incorporation of multiwall carbon nanotubes (MWCNTs) significantly enhanced the catalyst's specific surface area (from 251.2 to 611.3 m^2^/g) and facilitated the dispersion of Pd nanoclusters. Consequently, this catalyst exhibited a 22% increase in methane conversion compared to commercial catalysts within the temperature range of 700°C–850°C [60]. Azancot et al. investigated the impact of the preparation method (impregnation, precipitation, or a combination) on the metal‐support interaction of Ni Mg Al catalysts for SMR. Reducibility studies revealed that the impregnation‐coprecipitation method yielded the most favorable metal‐support interaction, effectively reducing NiO while maintaining highly dispersed Ni^0^ particles on the support surface. This optimal interaction led to higher methane conversion and hydrogen yield than catalysts prepared using only one method [61].

The structured shape of the catalyst can also present different results in catalytic activity performance. Zarei‐Jelyani et al. investigated the synergistic effects of Ni‐Co alloy catalysts supported on hollow spherical alumina that was developed in a previous study, as shown in Figure 2c. The hollow structure prevented metal particle aggregation during synthesis and catalysis. The Ni_2_‐Co1/H‐Al (hollow alumina support) catalyst (2:1 Ni: Co ratio) demonstrated the greatest methane conversion (90.72%) and hydrogen yield (93.82%) at 700°C. Characterization using fied emissionscanning electron microscopy (FESEM) and energy‐dispersive x‐ray spectroscopy (EDX) revealed reduced coke deposition on the hollow alumina‐supported catalyst compared to bulk alumina, suggesting its suitability for high‐temperature reactions [64].

From a structural perspective, the hollow sphere catalysts exhibited outstanding results attributed to their increased specific surface area and unvarying distribution of active sites on the support [71]. Salahi et al. study highlighted the significant influence of support type on catalytic performance. This conclusion was supported by ANOVA analysis, as can be seen in Figure 2d,e, which revealed the highest F values for support type (E) compared to other parameters like Ni loading (A), yttrium loading (B), temperature (C), and S/C molar ratio (D) [57]. Zarei–Jelyani enhanced the catalyst performance by incorporating cerium oxide as a promoter. Cerium's exceptional redox properties, particularly at lower temperatures, effectively reduce carbon formation on the catalyst surface. Despite introducing cerium loading as a new variable, statistical analysis still ranks support type as the third most influential parameter in SMR catalysts [66]. This finding offers a promising avenue for future research, particularly in exploring novel catalyst support types and structures that can enhance surface area and minimize coke formation.

Han et al. studied the impact of boron doping on Ni/MgAl_2_O_4_ catalysts. Their findings indicated that boron addition promoted nickel dispersion, leading to smaller nickel particles. Boron doping also facilitated incremental changes of chemisorbed oxygen content on the catalyst surface and thereby formed oxygen vacancies. These modifications suppressed nickel particle sintering and carbon formation during SMR, resulting in enhanced catalytic performance. The Ni‐B_0.5_/MgAl_2_O_4_ catalyst demonstrated a 90.2% methane conversion rate and 85% hydrogen yield at 700°C [68]. Meloni et al. conducted an interesting investigation into the potential of a Ni/CeO_2–_Al_2_O_3_ catalyst supported on electrified OB‐SiC foams.

By leveraging the Joule heating effect, they achieved uniform and efficient heating across the entire catalytic surface. This innovative approach showcased the remarkable versatility of the Ni‐based catalyst on OB‐SiC foams, resulting in an impressive conversion rate of approximately 99% and a hydrogen yield of around 85% at 720°C under atmospheric conditions [69]. Recent research advancements in Ni‐based catalysts for SMR have yielded promising results. These developments demonstrate a growing trend toward more efficient hydrogen production processes utilizing the SMR method.

**FIGURE 3:**
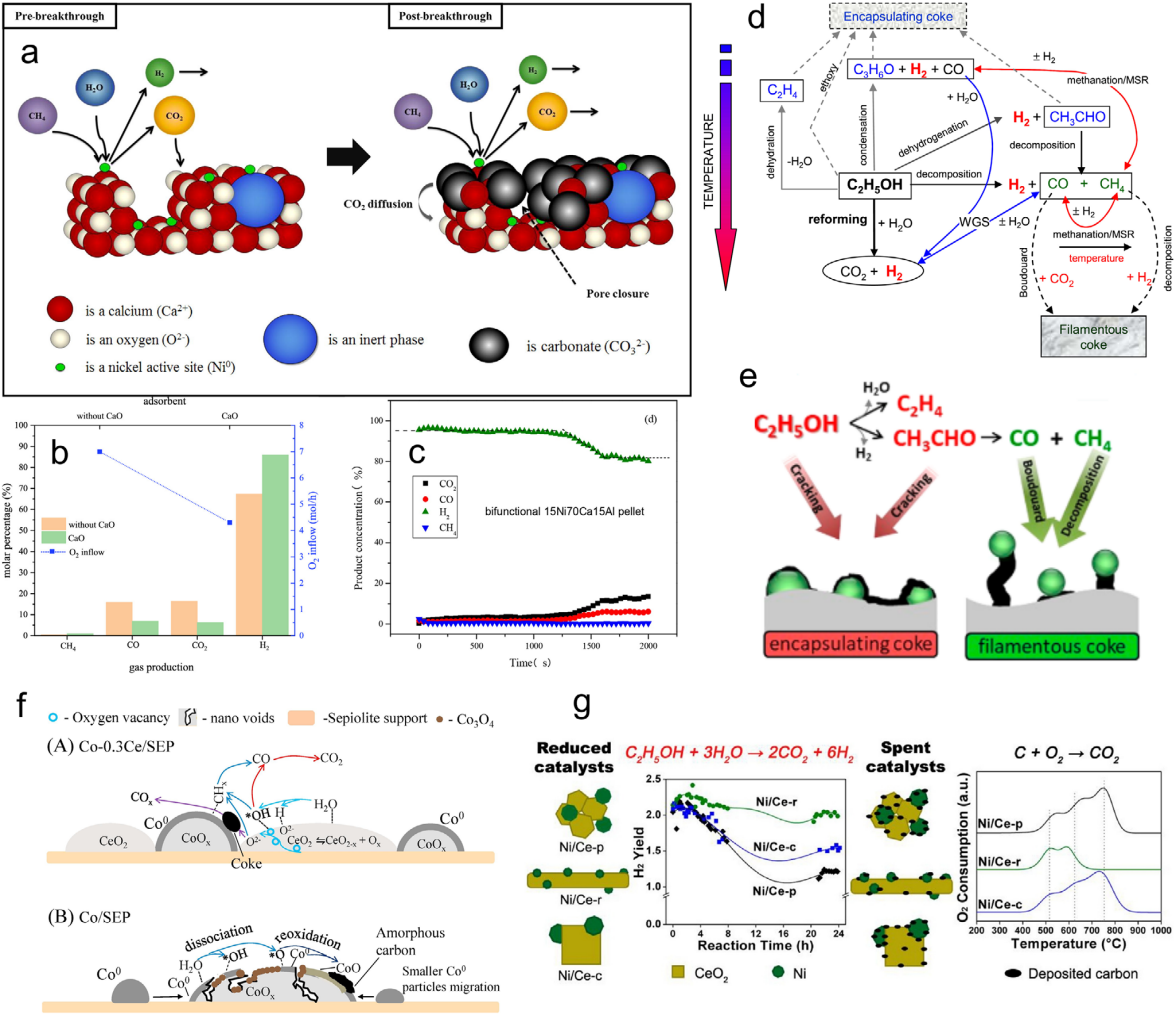
Mechanistic insights and catalytic performance in the SESMR and ESR processes. (a) The mechanism for CaCO_3_ densification in the SESMR process. Reproduced with permission [75]. Copyright 2014, Elsevier B.V. (b) Molar percentages of each gas present both with and without the addition of CaO, in the SESMR process. Reproduced with permission [76]. Copyright 2019, American Chemical Society. (c) The CO_2_ adsorption performance of sorbents and bifunctional materials at a temperature of 600°C. Reproduced with permission [77]. Copyright 2017, Elsevier B.V. (d) Plausible reaction mechanism of ESR. Reproduced with permission [74]. Copyright 2024, American Chemical Society. (e) Type of coke during ESR and its formation mechanism. Reproduced with permission [72]. Copyright 2024, Elsevier B.V. (f) The effect of Ce on the transformation of Co/SEP catalyst surface phase. Reproduced with permission [78]. Copyright 2019, Elsevier B. V. (g) H_2_ yield of Ni/Ce‐r (rod), Ni/Ce‐c (cubes), and Ni/Ce‐p (particles) and carbon deposits characterization by TPO profiles of carbon gasification. Reproduced with permission [79]. Copyright 2020, Elsevier B. V.

Currently, most researchers are trying to improve the performance of the SMR process while reducing the environmental impact of the process. Sorption‐enhanced steam methane reforming (SESMR) was introduced as a new SMR process with the CO_2_ absorbed in situ [72, 73]. CO_2_ capture adsorbents should have high CO_2_ adsorption capacity, stability, and durability. Among the common solid adsorbents, CaO‐based adsorbents derived from limestone and dolomite are promising due to their abundance and high CO_2_ capture potential [74]. Figure 3a illustrates the proposed mechanism of CO_2_ absorbed over a Ni‐based multifunctional catalyst supported by modified CaO. This scheme shows how the mechanism of the densification of CaCO_3_ after CO_2_ is absorbed into the CaO surface. From the study done by Phromprasit et al., we know that this mechanism over many cycles will reduce the methane conversion in the SMR process. The decline in performance was caused by the formation of CaCO_3_, which blocked the active nickel sites on the catalyst's surface [74]. Therefore, further research is needed to modify the absorbent to increase CO_2_ absorption without reducing the performance of hydrogen production in the SMR process. The formation of CaCO_3_ during sorption/desorption cycles is caused by the reaction between CaO and CO_2_ shown in Equation (3):

(3) $$\mathrm{CaO}+CO_{2}↔\mathrm{CaC}O_{3}$$

The addition of CaO‐based sorbent into a Ni‐based catalyst significantly increases the methane conversion rate. It reduces the CO and CO_2_ concentration, thereby raising the H_2_ concentration in the SMR process as shown in Figure 3b. Another study by Wang et al. also presents a similar result when the H_2_ concentration improves by the addition of CaO‐based sorbent from 80% to over 95% as shown in Figure 3c [72].

Li et al. developed a novel bifunctional catalyst for sorption‐enhanced SESMR hydrogen production. By strategically combining the phase transition characteristics of Ca‐Co‐O compounds and Ni‐Co alloys, they successfully mitigated the sintering of CaO, thereby enhancing the catalyst's durability. 1Ni_4.5_Co_4_._5_CaO catalyst exhibited exceptional catalytic performance in the SESMR reaction, achieving a hydrogen concentration exceeding 99.6% and complete methane conversion. Furthermore, the catalyst demonstrated remarkable stability over 50 cycles of operation, with minimal decline in hydrogen production and CO_2_ uptake capacity [70]. The study by Papalas et al. also that the bimetallic Ni‐Co oxygen carrier exhibited superior performance in hydrogen production, achieving high purity and yield while significantly improving energy recovery compared to monometallic Ni‐based materials in the SESMR coupled with chemical looping process. The catalyst maintained high‐purity hydrogen yield and auto‐thermicity over 22 cycles, demonstrating its durability for 30 h of operation [80].

The results of the research are getting closer to producing significant catalyst performance for the conversion of methane to hydrogen and performing in situ CO_2_ adsorption to make the SESMR process one of the options for blue hydrogen production. However, the trade‐off between methane conversion and CO_2_ uptake needs to be overcome with various strategies, especially the combination of bimetallic materials, which are currently giving promising results. Combined with previous findings related to the shape structure of the supported catalyst that has a significant influence on methane conversion, and utilizing absorbents such as CaO in the catalyst support will be an interesting thing to study in the future in the SESMR process. Toward connecting the gap of theoretical contribution and application, a work by Shen et al.’s marked the first demonstration of nickel‐based SiC catalytic materials for SMR under realistic scaling‐up conditions. Their novel monolithic SiC catalyst was tested in a fixed‐bed reactor at high gas hourly space velocities (10 000–25 000 h^−^^1^) under industrially relevant parameters, low feeding concentrations, and low temperatures/pressures, demonstrating the catalyst's efficient operation in harsh conditions and its potential for scaling up hydrogen production [81].

#### 2.1.2 Ethanol Steam Reforming

Hydrogen is a clean, versatile energy carrier with strong potential to replace fossil fuels. Thermochemical routes, using heat‐driven chemical reactions, can produce hydrogen from like natural gas, ammonia, or ethanol. While current production relies mainly on non‐renewable methane steam reforming, sustainable alternatives are needed. Ethanol steam reforming (ESR) offers a promising option, as ethanol can be derived from renewable sources such as sugarcane, cellulosic biomass, and corn. In addition, ethanol presents a series of advantages, since it is easier to store, handle, and transport in a safe way due to its lower toxicity and volatility [82, 83].

Different mechanistic schemes for ESR have been proposed to elucidate the real ethanol conversion mechanism on the catalyst surface. One of the proposed mechanistic schemes is shown in Figure 3d. The reaction steps primarily include (i) ethanol dehydrogenation to H_2_ and acetaldehyde followed by acetaldehyde decomposition to CH_4_ and CO; (ii) condensation of ethanol to acetone, H_2_, and CO; (iii) decomposition of ethanol to CH_4_, CO, and H_2_; (iv) methane steam reforming, and (v) water gas shift reaction [75]. Possible side reaction, ethanol dehydration to ethylene, may occur followed by ethylene polymerization to form coke. Ethanol conversion rate and hydrogen yield are greatly influenced by various parameters, including reaction temperature, water‐to‐ethanol ratio, reaction time, and catalyst [84].

Noble metal catalysts such as Pt, Pd, Rh, and Ru are well known for their high catalytic activity in the ESR due to their excellent ability of bond breaking and carbon deposition inhibition. Despite their high catalytic performance in ESR, the prohibitive cost of noble metals overshadows their potential use as active phases in catalysts. Consequently, in recent times, many studies have focused on non‐noble metal‐based catalysts, such as nickel, cobalt, and ceria, due to their low price and high catalytic activity. Despite its promising aspects, ethanol steam reforming faces several challenges. One of them is catalyst deactivation, which can occur due to coke deposition on the catalyst surface and metal particle sintering [85]. There are two distinct types of coke that have different effects on catalyst deactivation. The first type is encapsulated coke, which may be generated from the decomposition of ethylene and cracking of acetaldehyde and ethanol. The second type is filamentous coke, which is usually generated from methane decomposition and CO disproportionation (Boudouard reaction), as shown in Figure 3e. Encapsulated coke will block active sites and directly deactivate catalysts. Filamentous carbon typically has a limited effect on deactivation by partial blockage of catalyst pores when a high amount of coke is deposited. The presence of acidic sites on the catalyst promotes ethylene formation via dehydration of ethanol, which is a well‐known coke precursor. Additionally, acidic sites favor cracking reactions that yield coke as well [86]. Modification of the catalyst by the addition of basic oxides neutralizes the acidic sites and restrains coke formation [87, 88]. Addition of material with high oxygen storage ability and oxygen mobility through their redox behavior, such as ceria oxide and zirconia oxide, also help suppressing coke deposition through its combustion with oxygen atoms from the metal oxide lattice [89, 90].

Among non‐noble metal catalysts, Ni and Co are the most widely used active metals for ESR [91, 92, 93]. Rossetti et al. investigated the performance of Ni, Co, and Cu supported on SiO_2_ on ESR at a temperature range of 300°C–500°C. It was found that at 500°C, complete ethanol conversion was achieved for every catalyst except Cu/SiO_2_, with only 12% ethanol conversion [94]. In terms of H_2_ productivity, Ni/SiO_2_ showed the best performance with 1.3 ± 0.3 mol/min kg_cat_, followed by Co/SiO_2_, 0.95 ± 0.019 mol/min kg_cat_. In contrast, there is no H_2_ was obtained from Cu/SiO_2_. Moreover, methane is the only by‐product obtained with Ni/SiO_2_, whereas some acetaldehyde and ethylene were observed from Co/SiO_2_, which indicates the rate of C─C bond cleavage is higher for Ni/SiO_2_. In addition, Temperature‐programmed reduction analysis showed Ni/SiO_2_ has stronger metal‐support interaction, evidenced by a lower reducibility of the metal ion, leading to highest resistance to coking, mainly due to smaller Ni particle size.

The catalyst support plays an important role in the ESR process, not only promoting the interaction between the metal catalysts and the supports but also enhancing the dispersion of the metal on the support as well. Li et al. synthesized two Ni catalysts supported on ZrO_2_ by the impregnation method with different particle sizes [95]. The smaller ZrO_2_ particle (22.9 nm) was prepared by precipitation of zirconium oxychloride (Ni/ZrO_2_‐QP), meanwhile the larger ZrO_2_ particle (56.6 nm) was a commercial zirconia (Ni/ZrO_2_‐C). Both samples showed a typical IV‐type isotherm, indicating the presence of a mesopore framework with surface areas of 27.7 and 6.1 m^2^/g for Ni/ZrO_2_‐QP and Ni/ZrO_2_‐C, respectively. Additionally, the nickel particle size in Ni/ZrO_2_‐C is much larger than in Ni/ZrO_2_‐QP, which may result from the poor dispersion of nickel metal due to a small surface area and limited pore volume.

Furthermore, the H_2_‐TPR profile showed that Ni metal‐support interaction in Ni/ZrO_2_‐QP is stronger than Ni/ZrO_2_‐C. The presence of strong Ni metal‐support interaction would prevent the Ni metal from sintering, thus increasing the dispersion of Ni and the length of the interfacial boundary between Ni and ZrO_2_. The catalyst with a smaller particle size and higher metal dispersion, Ni/ZrO_2_‐QP exhibited a higher H_2_ yield of 79.9% compared to Ni/ZrO_2_‐C. In addition, coke formation in Ni/ZrO_2_‐QP was nearly five times lower than Ni/ZrO_2_‐C. Similar particle size dependency was also observed by another research group [96]. It was reported that the initial turnover frequencies (TOF) value of the smaller Ni particle is higher than the larger one. The same conclusion was reported for Co supported on carbon nanofiber catalyst. It was found that decreasing Co particle size increases the fraction of unsaturated Co surface (edge and corner sites), which are active for ESR, and thereby the TOF value increased [97].

Bimetallic catalysts, formed by combining two metals, have gained wide attention in energy applications due to their tunable physical and chemical properties, which are largely influenced by composition, preparation method, and structure. Zhao et al. found out that monometallic catalysts, Ni/Al_2_O_3_ and Co/Al_2_O_3,_ were active in ESR, but their performance, both in terms of conversion of ethanol and yield of H_2_ were inferior compared to the bimetallic catalyst, Ni‐Co/Al_2_O_3_ [98]. In the same study, the influence of preparation method on the performance of Ni‐Co supported on Al_2_O_3_ was investigated. Simultaneous co‐impregnation of the two metals (Ni‐Co/Al_2_O_3_) was compared to the sequential addition of Ni and Co to Al_2_O_3_, in either order. The catalyst was denoted as Co/Ni/Al_2_O_3_ (Ni was added first) and Ni/Co/Al_2_O_3_ (Co was added first). H_2_ chemisorption at 25°C revealed that the dispersions of Ni‐Co/Al_2_O_3_, Co/Ni/Al_2_O_3_, and Ni/Co/Al_2_O_3_ were 31.5%, 29.1%, and 28.0%, respectively. Interestingly, at temperatures exceeding 350°C, the ethanol conversion and H_2_ yield followed the order Ni‐Co/Al_2_O_3_ > Co/Ni/Al_2_O_3_ > Ni/Co/Al_2_O_3_, which is the same order as the metal dispersion. In addition, the thermogravimetric results showed that the Ni‐Co/Al_2_O_3_ catalyst showed better carbon resistance, which could result from high metal dispersion. The author also mentioned the catalyst could produce hydrogen spillover, which could promote surface cleaning through the reaction between carbon deposits on the surface and hydrogen. For a fair comparison, we outline several various non‐noble metal catalysts for ESR shown in Table 2.

**TABLE 2:** Catalytic performance of various non‐noble metal catalysts in ESR within 2018–2024.

Catalyst	Temp (°C)	S/C ratio	Conversion (%)	H_2_ yield (%)	H_2_ selectivity (%)	Coking rate (mg_c_/g_cat_.h)	Stability test (X_EtOH_) ^a^	Space velocity	Ref
10Co/α‐Al_2_O_3_	500	12	100		96	—	24 h (100%)	60 L g^−1^ h^−1^	[99]
17Co/α‐Al2O3	500	6	86	64	—	—	—	51 700	[100]
NiAl_2_O_4_	550	3	100	42	—	—	—	6 000 L kg^−1^ h^−1^	[101]
8.8Ni/6.8La_2_O_3_‐Al_2_O_3_	650	3	100	73	—	—	200 h (97%)	2 773 h^−1^ (WHSV)	[102]
Ni/Ce	550		33	18	—	—	—		[103]
Ni/La‐Ce	550		100	70	—	—	60 h (90%)	2.26h^−1^ (WHSV)	[103]
10Ni/CeMgAl	500	6	90.6	—	66.9	0.72	50 h (73.9%)	10 h^−1^ (LHSV)	[104]
9Ni1Co/CeMgAl	500	3	93.9	—	69	0.5	100 h (93.9%)	10 h^−1^ (LHSV)	[105]
5Ni/CZ‐IM	550	6	57.21	—	71.09	62.66	—	10 619 h^−1^ (GHSV)	[106]
5Ni/CZ‐OP	550	6	99.99	—	74.85	1.43	—	10 619 h^−1^ (GHSV)	[106]
Ni–Mo_2_C–Al_2_O_3_	700	10	100	45		2.2	—	200 000 mL h^−1^ gcat^−1^	[107]

^a^ X_EtOH_ = Conversion of ethanol

It is well known that acidic sites on catalyst support such as Al_2_O_3_ and ZrO_2_ can initiate dehydration and polymerization reactions that lead to coke formation, thus may deactivate the catalyst. The most common strategy to improve the neutrality of acidic supports is by incorporation of basic materials such as alkali, alkaline earth oxide, or lanthanide oxides. Choong et al. reported that the addition of Ca on Ni/Al_2_O_3_ has a positive effect on catalyst stability [88]. Ethanol conversion declined after 12 h of operation over the catalyst without Ca, 10Ni/Al_2_O_3_. In contrast, 3 wt.% Ca‐incorporated catalyst, 10Ni/3Ca‐Al_2_O_3_ remains active with 100% ethanol conversion and 90% selectivity to H_2_ during the whole test for 24 h. However, further increasing Ca loading to 5 wt.% is detrimental to catalyst stability. The deactivation occurs after 10 h of reaction, and ethanol conversion drops to 30%. Evidently, incorporation of Ca at a certain amount plays a role in the improvement of catalytic stability.

Furthermore, due to its ability to capture CO_2_, CaO is used in sorption‐enhanced ethanol steam reforming, a hybrid process of CO_2_ capture and ESR performed simultaneously in a single reactor. Based on Le Chatelier's principle, the removal of CO_2_ during ESR will shift the reaction equilibrium toward H_2_ [108]. Ni/Al/Ca‐85.5, where 85.5 represented the mass content of CaO, exhibited superior catalytic activity compared to Ni/Al_2_O_3_. Ni/Al/Ca‐85.5 trapped CO_2_ effectively and produced high H_2_ purity of about 96% during 23 min of reaction. In contrast, due to the lack of sorption enhancement, the Ni/Al_2_O_3_ only achieved a H_2_ purity of about 73.8% [109]. Alcohol steam reforming, which involves many redox reactions such as water activation, is often identified as a rate‐limiting step reaction. To enhance the catalytic performance and stability, materials with superior redox properties have garnered significant attention due to their ability to facilitate efficient water activation and promote the oxidation of carbonaceous deposits [110, 111]. Among these, ceria stands out due to its exceptional oxygen storage and release capabilities by readily altering its oxidation states between Ce^4+^ and Ce^3+^ [112]. The mobile oxygen released from the lattice can promptly react with carbonaceous species, preventing their accumulation on the metal surface and thereby suppressing catalyst deactivation. Li et al. reported that the incorporation of CeO_2_ on Ni/SBA‐15 could reduce Ni particle size and enhance the Ni dispersion [113]. 1CeNi/SBA‐15, a catalyst with a Ce/Ni atomic ratio of 1 showed superior catalytic activity compared to Ni/SBA‐15, a catalyst without the addition of ceria. Complete ethanol conversion with hydrogen selectivity of 97% was achieved by 1CeNi/SBA‐15. In addition, it also exhibited great catalytic stability as it maintained 90% ethanol conversion even after 50 h. Moreover, based on thermogravimetric analysis, temperature‐programmed oxidation, and X‐ray diffraction analysis of the spent catalyst, it can be concluded that 1CeNi/SBA‐15 possesses a lower amount of coke deposition compared to Ni/SBA‐15.

The positive effect of ceria contribution was also observed on cobalt‐supported sepiolite (Co/SEP) [78]. The carbon conversion and H_2_ yield increased from 53.5% and 33.6% over Co/SEP to 90.8% and 69.1% over Co‐0.3Ce/SEP, a catalyst with a Ce/Co weight ratio of 0.3. The superior redox properties and higher hydrophilicity of CeO_2_ enabled it to efficiently adsorb and dissociate water molecules in the Co‐0.3Ce/SEP catalyst during the reforming process, facilitating the mobility of surface *O/*OH, consequently enhancing the ESR activity and tremendously alleviating surface re‐oxidation of metallic Co particles. Thus, there is an adequate supply of oxygen‐free radical species that rapidly consume carbon species on the surface of the metal, helping maintain a clean metal surface as shown in Figure 3f.

Additionally, the lower surface area of the Co/SEP catalyst, which is detrimental to mass transfer and the diffusion of intermediate products, also contributes to the lower catalytic activity of Co/SEP. Furthermore, even though the overall effect of the shape of ceria particles on catalytic activity is not entirely clear, it is widely accepted that CeO_2_ with different morphologies such as nanorods, nanocubes, and nanopolyhedra may exhibit different oxygen mobilities due to the exposition of different lattice planes. There are more oxygen vacancies on (110) and (100) planes, which are favorable for ESR. Polycrystalline ceria nanoparticles usually consist of octahedra shapes, which mainly expose (111) planes, whereas nanorods expose (110) and (100), and nanocubes mainly expose (100) [114, 115]. Vecchietti et al. studied the ethanol surface reaction over CeO_2_ nanooctahedra (CeO_2_‐NO), which mainly exposes (111) planes, and CeO_2_ nanocubes (CeO_2_‐NC), which are dominated by (100) planes. It was found that the production of H_2_ is 2.4 times higher on CeO_2_‐NC than on CeO_2_‐NO, even though the ethanol conversion is lower for CeO_2_‐NC [116]. In addition, CeO_2_‐NO is more selective toward ethylene rather than H_2_. These results indicate that the CeO_2_(111) surface is more active for ethanol dehydration, producing ethylene. Whereas the CeO_2_(100) surface seems to be better at breaking the C‐C bond than (111) and is more selective toward H_2_ and CO_2_. Furthermore, the effect of ceria morphology (particles, rods, and cubes) on the carbon deposition during ESR over Ni/CeO_2_ was also studied by Araiza et al. [79] Nickel supported on ceria nanorods (Ni/Ce‐r) revealed the highest hydrogen yield in the ESR reaction at 550°C for 24 h with the lowest amount of carbon deposits, as can be seen in Figure 3g. Other studies related to ESR over non‐noble metal catalysts in the last six years can be seen in Table 2.

### 2.2 Photocatalytic H_2_ Production

Photocatalytic H_2_ production has gained significant attention since the pioneering work of Fujishima and Honda in 1972 [117], a photoelectrochemical water splitting into O_2_ and H_2_ using a TiO_2_ photoanode coupled with a Pt cathode under UV light irradiation. Since then, extensive research efforts have been devoted for the development of efficient, inexpensive, and wide‐spectrum‐responsive photocatalysts with high specific surface area. Being inspired by the photosynthesis process in nature [118], a heterogeneous photocatalytic system employing semiconductor powders suspended in solution has also been extensively studied. This system basically uses the simple configuration of one reaction chamber, so separation of the reaction products may be further required. Meanwhile, the photoelectrocatalytic system enables ease of product separation and additional external voltage as needed to manipulate the reaction mechanism and selectivity.

In principle, there are common properties that need to be possessed of photocatalytic materials to facilitate the redox reaction regardless of the system type used. Upon photoexcitation, the generated electron and hole pairs are separated and transported to the catalyst surface to induce the redox reaction. Ideally, these photoexcited electrons and holes are used up in the reaction, otherwise, they would be lost through charge recombination. Water splitting into H_2_ and O_2_ is a thermodynamically unfavorable process that requires a potential of 1.23 eV [118, 119]. Therefore, in photocatalytic water splitting, the photocatalysts should have a band gap larger than 1.23 eV and suitable band edge potential to facilitate the reaction. The conduction band edge of the semiconductor has to be positioned at a more negative potential than that of the H_2_ evolution potential (−0.41 V relative to a normal hydrogen electrode at pH 7), while the valence band has to be more positive than that of the oxidation potential of water to produce O_2_ (+0.82 V relative to a normal hydrogen electrode at pH 7) [118]. Strong light absorption, excellent charge separation, and high redox capabilities are essential to photocatalytic performance. In this regard, some strategies have been developed, such as band gap engineering to reduce the band gap, altering the position of band edges, and cocatalyst integration [120, 121, 122].

**FIGURE 4:**
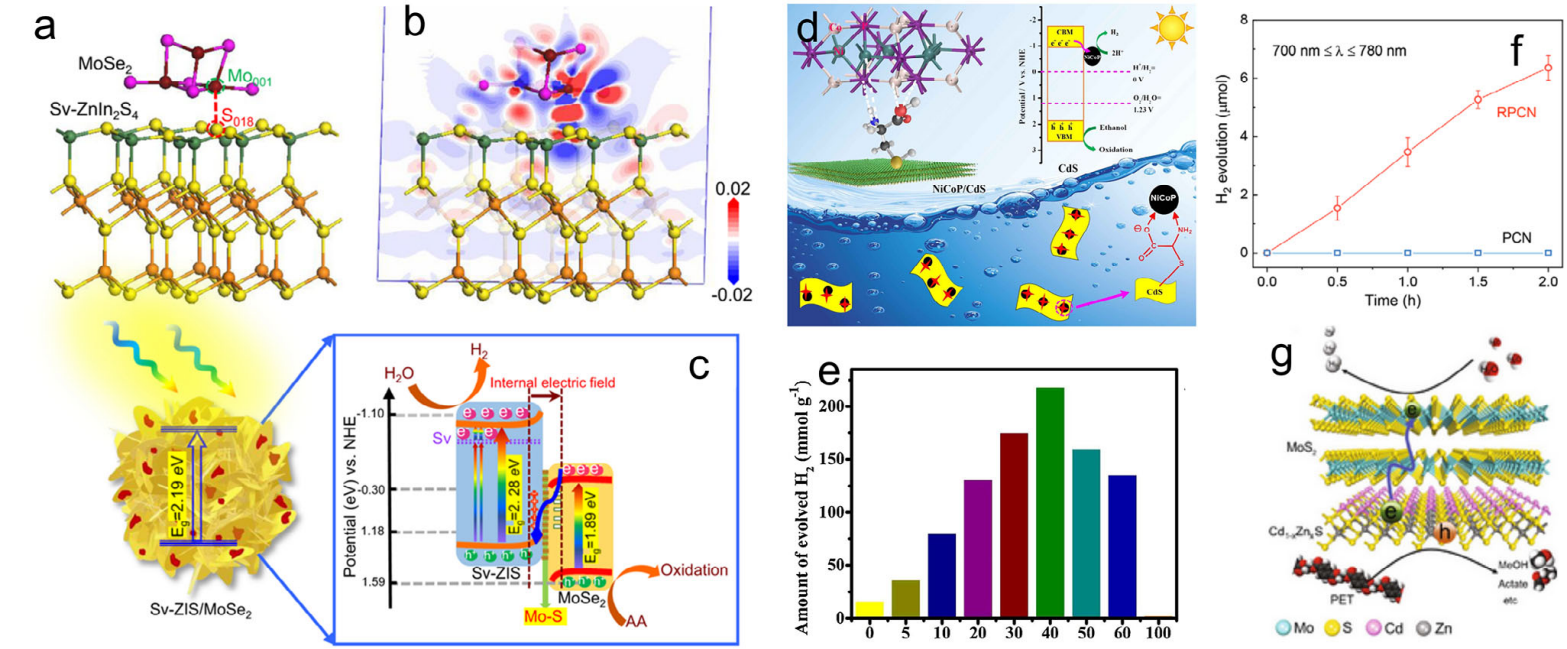
Development of non‐noble metal‐based photocatalytic H_2_ production. (a) The optimized structure, (b) the charge density difference, and (c) Photocatalytic reaction of S_v_‐ZnIn_2_S_4_/MoSe_2_ heterostructure under light irradiation. Reproduced with permission [127]. Copyright 2021, Springer Nature. (d) Photoinduced charge transfer for L‐cysteine‐capped NiCoP/CdS photocatalyst and (e) the rate of H_2_ evolution with different amounts of NiCoP loaded on CdS under visible light irradiation (λ > 420 nm). Reproduced with permission [128]. Copyright 2020, Elsevier B.V. (f) H_2_ evolution of carbon/potassium‐doped RPCN under NIR irradiation. Reproduced with permission [120]. Copyright 2021, Wiley‐VCH GmbH. (g) H_2_ coupled with degradation of PET plastic over 2D/2D MoS_2_/Cd_x_Zn_1‐x_S photocatalyst. Reproduced with permission [129]. Copyright 2020, Wiley‐VCH GmbH.

Exploiting the entire solar spectrum and using less‐expensive non‐noble metal‐based catalysts have been the main driver of progress in the photocatalytic field, particularly for large‐scale applications. The following description shows some recent developments of non‐noble metal photocatalysts for hydrogen production, particularly in utilizing efficiently the broadband full‐spectrum of solar light irradiation. Considering the limitations of single‐component semiconductor materials, the construction of heterojunctions may be an effective strategy to enhance photocatalytic activity [122, 123, 124]. Heterojunction with suitable band alignment can lead to better light absorption and promote rapid charge separation and/or transfer. Compared to the conventional type II heterojunction, the Z‐scheme heterojunction resembling the natural photosynthesis process, has attracted much interest due to the stronger redox capacity, in which at the heterostructure interface, the photoexcited electrons in one photocatalyst with lower conduction band minimum can recombine with the photoexcited holes in another photocatalyst with higher valence band maximum (VBM), so that more oxidative holes and reductive electrons can be maintained in different counterparts, which improve the photocatalytic efficiency [125, 126]. In case of S_v_‐ZnIn_2_S_4_/MoSe_2_ heterostructure shown in Figure 4a–c, the synergy among interfacial Mo‐S chemical bond, internal electric field, and S vacancies could induce the Z‐scheme charge transfer mechanism, optimizing the hydrogen evolution rate of 63.21 mmol.g^−1^.h^−1^ under visible light (λ > 420 nm) and retenting H_2_ evolution about 90.5% after 20 h of 5 photocatalytic test cycles [127].

On the other hand, to enable charge separation and maintain the stability of multicomponent catalysts, strong binding at the heterojunction of different catalyst materials has also been considered, such as using linker molecules. As shown in Figure 4d,e [128], L‐cysteine capped NiCoP/CdS photocatalyst demonstrates efficient spatial charge separation and transfer by the covalent bond formed via L‐ Cys between NiCoP and CdS, exhibiting an H_2_ evolution rate of 218 mmol.g^−1^.h^−1^ under visible light irradiation and a stability of 192 h. Further application of photocatalysis beyond the visible light region has been a formidable challenging. A strategy to use carbon/potassium‐doped red polymeric carbon nitride (RPCN) has been reported to successfully produce H_2_ (140 µm.g^−1^.h^−1^) under NIR irradiation as shown in Figure 4f [120]. Besides extending the spectral response, attempts to couple H_2_ production with other oxidation reactions have been made, such as those in photocatalytic reforming of lignocellulose to H_2_ [130]. Furthermore, coupling the hydrogen evolution with the degradation of polyethylene terephthalate plastic, as shown in Figure 4g, has been reported by utilizing a 2D/2D MoS_2_/C_x_Z_1‐x_S photocatalyst, which may indicate the possibility of simultaneously addressing the problems of plastic pollution and the demand for sustainable clean energy [129]. Recent work on several developments of photocatalytic H_2_ production is depicted in Table 3.

**TABLE 3:** Some recent reports on photocatalytic hydrogen production.

Photocatalyst	Solution/sacrificial agent	Light source	H_2_ production (g^−1^∙h^−1^)	AQY (%)	Benchmark (g^−1^∙h^−1^)	Ref.
2D/2D 15% FeSe_2_/g‐C_3_N_4_	0.15/0.35 mol/L Na_2_S/Na_2_SO_3_	Visible light (λ > 420 nm)	1 655.6 µmol	10.89	Pt/g‐C_3_N_4_: ∼1 700–2 000 µmol [131]	[132]
0.3 CNS/g‐C_3_N_4_	10.0 vol % triethanolamine	Visible light (λ > 420 nm) Infrared (λ > 800 nm)	752.8 µmol 0.32 µmol	0.04 4.38	Pt/g‐C_3_N_4_: ∼800–1 000 µmol [133, 134]	[135]
2D/2D 4.3 wt.% MoS_2_/Cd_0.5_Zn_0.5_S	Supernatant of 60 mL 10 m NaOH + 1.5 g PET	AM 1.5G	15.90 mmol	15.6 at 420 nm [136]	Pt/CdZnS: ∼16–20 mmol [137]	[129]
2D/2D β‐NiS‐decorated CdS/α‐Fe_2_O_3_	0.25 m Na_2_S /Na_2_SO_3_	Visible light (λ > 420 nm)	45 mmol	46.9	Pt/CdS: ∼45–55 mmol [138]	[122]
4 wt.% ReS_2_/Zn_0.3_Cd_0.7_S	Na_2_S and Na_2_SO_3_ aqueous	Visible light (λ > 420 nm)	92.45 mmol	20.95	Pt/ZnCdS: ∼90–100 mmol [139]	[140]
3% Ni‐doped Mn_0.5_Cd_0.5_S	0.35 M Na_2_S/0.25 M Na_2_SO_3_	Visible light (λ > 420 nm)	108.3 mmol	23.4 at 400 nm [141]	Pt/CdS: ∼100–120 mmol [142]	[121]
0D/1D W_18_O_49_/CdS	20 vol% lactic acid	Visible light (λ > 410 nm)	55.24 mmol	40.06	Pt/CdS: ∼55–65 mmol [143]	[2]
S_v_‐ZnIn_2_S_4_/MoSe_2_	0.1 m ascorbic acid	Visible light (λ > 420 nm)	63.21 mmol	76.48	Pt/ZnIn_2_S_4_: ∼60–70 mmol [144]	[145]
Carbon/potassium‐doped red polymeric carbon nitride with ∼3 wt.% Pt as cocatalyst	10 vol% triethanolamine	500 nm ≤ λ ≤ 780 nm NIR (700 nm ≤ λ ≤ 780 nm)	640 µmol 140 µmol	13.00 0.84	Pt/g‐C_3_N_4_: ∼500–700 µmol [146]	[120]
40 wt.% L‐cysteine capped NiCoP/CdS	ethanol‐water mixture (20 vol. %)	Visible light (λ > 420 nm)	218 mmol	76.3	Pt/CdS: ∼200–220 mmol [147]	[128]

### 2.3 Electrocatalytic H_2_ Production

Apart from being stimulated by light, water splitting to produce H_2_ can also be generated by forcing electricity through an electrochemical cell. For the cathodic HER, Pt has been well known as an ideal electrocatalyst owing to a low overpotential close to zero and a Tafel slope of about 30 mV/dec [148, 149]. The overpotential value refers to the additional potential required to overcome the energetic barrier for the charge transfer and to drive the HER at a certain rate (typically 10 mA/cm^2^). The lower overpotential is desirable due to the smaller energy barrier, which leads to a rapid charge transfer rate and subsequently a stronger catalytic HER ability. Determined at low overpotential, the Tafel slope is the change value of overpotential for every ten times increase of current density, considered an essential indicator in evaluating catalyst performance because it enables us to infer the mechanism occurring on the catalyst surface based on its value [150]. Basically, HER constitutes a two‐step process, i.e., the adsorption of H_2_O or H^+^ species on the cathode surface (called the Volmer step) and the chemical desorption of H_2_ (Tafel step) or the electrochemical desorption of H_2_ (Heyrovsky step) from the cathode [41, 149, 150, 151]. For good HER performance, electrocatalysts on the cathode should have a large number of active sites with optimal electron density to generate moderate bonding strength with adsorbed H atom intermediate (Sabatier principle) for a smooth adsorption or desorption process, a low charge transfer resistance across the interface as well as with the bottom electrode material, and stability in the electrolyte medium [41, 151, 152]. The smaller value of the Tafel slope indicates that the catalysts achieve a certain current density at a lower overpotential, implying a faster charge transfer rate and better HER kinetic performance. Moreover, the Tafel slope exhibits an apparent pH dependency, where the higher pH leads to a higher value of the Tafel slope due to the unfavourable hydrogen adsorption on the catalyst surface and thus impedes the HER [153]. At neutral and even alkaline conditions, Pt catalysts exhibit significantly lower HER activity compared to acidic conditions due to the sluggish kinetics of the water dissociation step, which is the rate‐determining step in non‐acidic media [28, 154, 155]. In this regard, development of non‐noble metal‐based catalysts possessing good water‐dissociation capability has demonstrated the potential to challenge the performance of Pt‐based catalysts [156, 157]. Therefore, low‐cost electrocatalysts with the ability to work effectively in any (acid, alkaline, and neutral pH) environment are vital for practical application.

**FIGURE 5:**
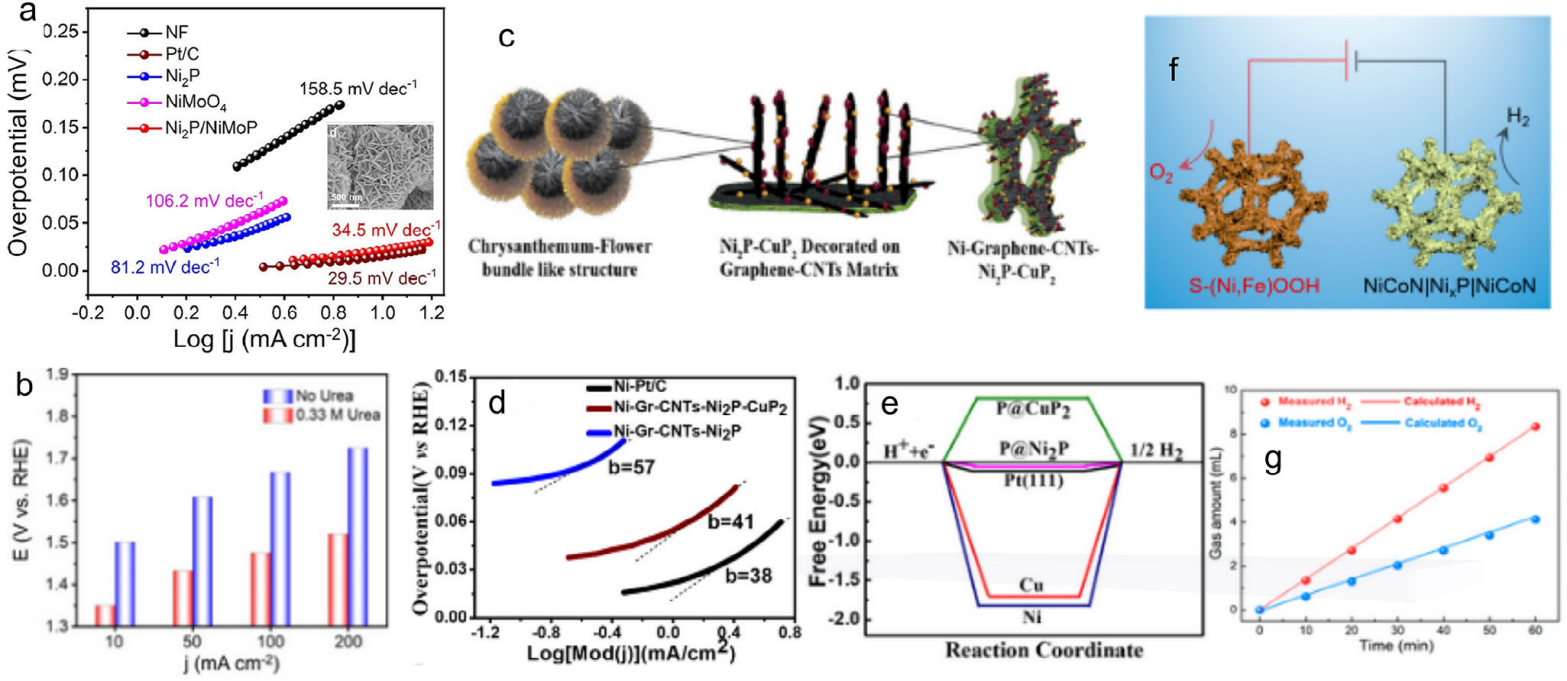
Development of non‐noble metal‐based electrochemical H_2_ production. (a) Tafel slopes of various catalysts with the inset of an SEM image of Ni_2_P/NiMoP and (b) the related electrolyzer cell voltage required to reach different current densities for urea electrolysis and water electrolysis. Reproduced with permission [159]. Copyright 2021, Elsevier B.V. (c) Chrysanthemum‐flower‐like bundle structure of hierarchical Ni‐graphene‐CNTs‐Ni_2_P−CuP_2_ heterostructure, (d) Tafel plots, and (e) free energy diagram of Ni_2_P−CuP_2_ heterostructure surface and Pt (111) reference for HER. Reproduced with permission [161]. Copyright 2021, American Chemical Society. (f) Seawater electrolyzer using NiCoN|Ni_x_P|NiCoN and S‐(Ni,Fe)OOH as the cathode and anode, respectively, and (g) gaseous production at a fixed current density of 20 mA/cm^2^. Reproduced with permission [162]. Copyright 2020, American Chemical Society.

For the large‐scale practical application of electrochemical water splitting, efforts have been made to develop cost‐effective and high‐performance electrocatalysts. Although noble metals offer excellent catalytic activity, their associated high cost and rarity limit their widespread adoption, prompting the search on electrocatalysts with a lesser quantities of noble metals or even not at all without compromising the performances. For example, 5.2 wt.% Rh‐MoS_2_ nanocomposites have been reported to exhibit superior electrochemical HER with a low overpotential of 47 mV at the current density of 10 mA/cm^2^ and a small Tafel slope of 24 mW/dec [158]. This superior performance is possibly caused by the fast hydronium ion capture at the strong H‐adsorbed component Rh to become the absorbed H atom and then its migration to the quick H_2_‐desorbed component MoS_2_ surface, which eventually becomes H_2_ gas. In the case of non‐noble metals‐based catalysts, an efficient Ni_2_P/NiMoP heterojunction electrocatalyst with a hierarchical architecture has been grown uniformly on a nickel foam substrate [159]. As shown in Figure 5a,b, this heterojunction electrocatalyst exhibited HER activity in alkaline solution with a low overpotential of 22 mV at 10 mA/cm^2^ and a low Tafel slope of 34.5 mV/dec, and the electrolyzer employing Ni_2_P/NiMoP as a bifunctional catalyst simultaneously for the urea (instead of oxygen) oxidation reaction showed a current density of 10 mA/cm^2^ at a cell voltage of 1.35 V with long term durability of 8h. Moreover, Fe_2_P/Co_2_N porous heterostructure arrays have been reported as non‐noble bifunctional electrocatalyts for both HER and OER simultaneously, displaying an overall water splitting activity with the required voltage of 1.561 V to attain 100 mA cm^−2^ with durability over 120 h for alkaline water electrolysis [160].

A chrysanthemum‐flower‐like bundle structure of hierarchical Ni‐graphene‐CNTs‐Ni_2_P−CuP_2_ heterostructure illustrated in Figure 5c has been reported to have an HER overpotential of 12 and 32 mV in acidic and alkaline solution, respectively, and an electrolyzer cell voltage of 1.45 V in alkaline solution, all at a current density of 10 mA/cm^2,^ with stability of at least 40 h for water splitting [161]. Here, as depicted in Figure 5d,e, the Tafel slope for HER in acidic solution was found to be 41 mV/dec, and the free energy diagram of P atom at the Ni_2_P side (P@Ni_2_P) shows a very small negative free energy even compared to that of the reported Pt (111) surface, indicating an excellent catalytic active site for HER. Additionally, a 3D nanohybrid of a 2D/2D/2D TiVCT_x_/MoS_2_‐CNT electrocatalyst has been developed and, in acidic solution, demonstrated hydrogen evolution activity with an overpotential of 180 mV at 10 mA/cm^2^ and an electrochemical durability of up to 17 h [163]. Furthermore, in single‐atom catalysts, Mo single atoms anchored on the Cr top site of 1T‐CrS_2_ metallic basal plane via S atom have been reported to induce tip effect with an enhanced electric field surrounding Mo atoms and an almost zero hydrogen adsorption free energy, exhibiting both superior HER performance and high stability to those of the 1T‐CrS_2_ [164].

For practical electrochemical hydrogen production, the direct utilization of seawater has emerged as an attractive research direction owing to no dependency on high‐purity water. In this regard, the NiCoN|Ni_x_P|NiCoN electrode exhibited HER activity with an overpotential of 165 mV at 10 mA/cm^2^ in natural seawater electrolyte [162]. For the seawater electrolyzer using NiCoN|Ni_x_P|NiCoN and S‐(NiFe)OOH as the cathode and anode, respectively, the gaseous production detected at a fixed current density of 20 mA/cm^2^ showed the volume ratio of the detected H_2_ and O_2_ gases close to 2:1, and the gas amounts matched well with the calculated results, as shown in Figure 5f,g. This seawater electrolyzer also exhibited no chlorine evolution (a competitive process with the oxygen evolution reaction or OER) on the anode and decent stability in natural seawater owing to the good corrosion resistance of the Ni_x_P skeleton and the anticorrosive NiCoN nanoparticles. Recently, the use of CuCo_2_S_4_ nanowires has demonstrated HER performance with overpotentials of 91.2 mV at 10 mA cm^−2^ in 1 m seawater KOH [165]. Despite its practicality, some other challenging issues should also be considered carefully, such as the slow kinetics of the OER (another half‐reaction process considered a rate‐limiting step in water splitting) and the possibility of precipitate formation on the cathode [159, 166]. For real applications, electrochemical water splitting may be powered by renewable energy resources like solar and/or wind for green hydrogen production. The advancement of solar‐to‐hydrogen and wind‐to‐hydrogen conversion technologies offers a clean and renewable energy carrier with no use of fossil fuels and paves the way for the future sustainable hydrogen fuel community.

In terms of other advancements, a high‐performance anion exchange membrane water electrolyzer (AEMWE) also shows promising performance with flexible WS_2_ superstructures as a high‐performance non‐noble metal cathode catalyst for HER in AEM electrolysis. Xie et al. show that conventional WS_2_ suffers from sluggish alkaline kinetics and poor durability under industrial current densities, but the engineered 3D superstructure, with expanded interlayer spacing, abundant stepped edge defects, and bond‐free van der Waals interactions, maximizes exposure of active sites, enhances charge transfer, and provides exceptional mechanical flexibility to withstand gas—liquid stresses [167]. Electrochemically, the WS_2_ superstructure achieved overpotentials as low as 205 mV at 500 mA cm^−2^ and 264 mV at 1 000 mA cm^−2^, outperforming Pt/C under high‐current operation. In a practical AEM electrolyzer (IrO_2_ anode), it delivered 1 A cm^−2^ at 1.70 V with remarkable stability over 1 000 h, showing a degradation rate of 9.67 µV h^−2^. These results establish WS_2_ superstructures as durable, scalable, and efficient HER catalysts for industrial hydrogen production.

Solid oxide electrolysis cells (SOECs) have also shown promising technology, such as reported by Han et al. in 2024 [168]. This work presents an interface‐engineering strategy to construct multi‐scale self‐assembled NiFe layered double hydroxide (NiFe‐LDH) heterostructures as highly active and durable OER catalysts. By inducing controlled interfacial interactions with conductive carbon substrates, the NiFe‐LDH nanosheets are uniformly distributed, creating vertically oriented arrays with enlarged surface area, abundant exposed active sites, and optimized electron pathways. The heterostructures also exhibit robust multi‐scale hierarchical porosity, which facilitates electrolyte diffusion and bubble release during OER at high current densities. Electrochemical evaluation demonstrates that the engineered NiFe‐LDH achieves an overpotential of 206 mV at 10 mA cm^−^^2^ and maintains only 269 mV at 500 mA cm^−^^2^, surpassing benchmark IrO_2_ catalysts in both activity and stability. The catalyst also sustains long‐term durability under industrial‐level current operation. These findings highlight interface engineering as a powerful tool to translate NiFe‐LDH catalysts into practical, scalable OER electrocatalysts for sustainable hydrogen production [168].

## 3 Advanced Spectroscopies for HER Characterization at the Nanoscale

Recent breakthroughs in nanoscale spectroscopic methodologies have transformed the mechanistic understanding of HER by enabling direct visualization of charge transfer, surface intermediates, and dynamic reaction pathways under operando conditions. In the following section, we provide an overview in the optical response and carrier dynamics governing HER activity using ultrafast pump–probe spectroscopy. Complementarily, nanoscopic electrochemical probes and scanning probe‐based techniques offer spatially resolved interrogation of active sites, local conductivity, and catalytic heterogeneity during electrocatalytic HER. Beyond electrochemical systems, advanced vibrational and synchrotron‐based spectroscopies have also emerged as indispensable tools for assessing thermocatalytic pathways in processes such as SMR, enabling real‐time tracking of surface adsorbates, carbon evolution, and catalyst restructuring under harsh reaction environments.

### 3.1 Ultrafast Pump‐Probe Spectroscopy for Carrier Dynamics in HER Cataylsts

**FIGURE 6:**
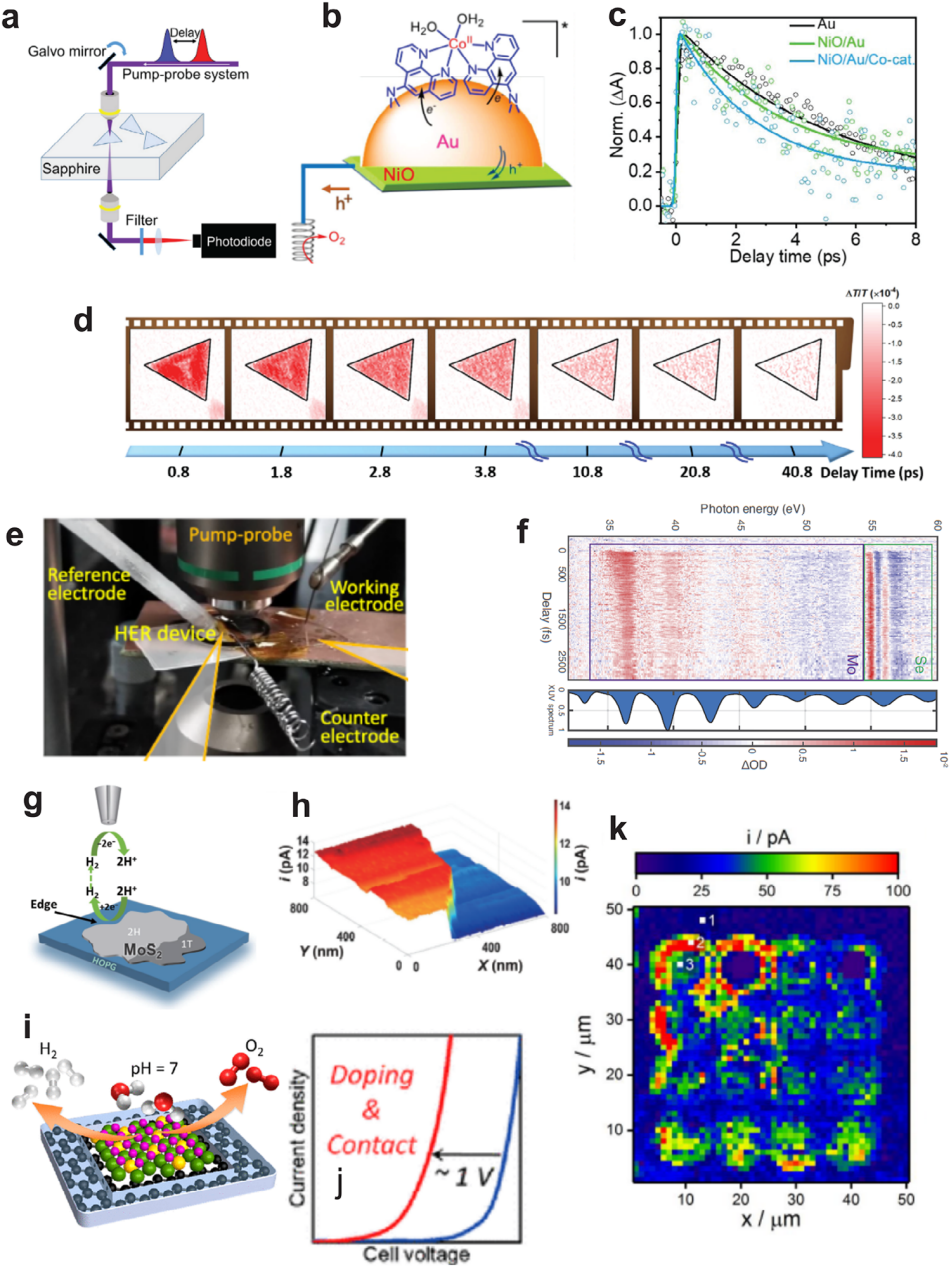
Advanced spectroscopic characterization of photo‐electrocatalytic systems for hydrogen evolution. (a) General schematics of an ultrafast pump‐probe spectroscopy. Reproduced with permission [180]. Copyright 2022, Wiley‐VCH GmbH. (b) Photosystem structure used for the photo‐electrocatalytic H_2_ evolution and (c) kinetic traces of Au, NiO/Au, NiO/Au/Co‐catalyst, extracted at 490 nm from the TAS measurement. Reproduced with permission [181]. Copyright 2024, Springer Nature. (d) UPPM images of the transient transmittance change ΔT/T from 0.8 to 40.8 ps of t‐MoS_2_. Reproduced with permission [180]. Copyright 2022, Wiley‐VCH GmbH. (e) In situ probing close‐up of HER devices under time‐resolved liquid cell microscope (TR‐LCM) geometry. Reproduced with permission [182]. Copyright 2022, ACS Publications. (f) ATAS measurement of a few‐layer MoSe_2_ sample where the optical response is elucidated due to electronic transitions from the Mo 4*p (*Se 3*d)* states plotted in the purple (green) frame. Reproduced with permission [183]. Copyright 2024, National Academy of Sciences. (g) HER monitoring of MoS_2_ edge on the substrate generation/tip collection (SG/TC) mode at 1T‐2H MoS_2_ nanosheets and (h) 3D color map indicating the transition of two contrasting regions at the edge of semiconducting MoS_2_ nanosheets. Reproduced with permission [184]. Copyright 2023, Springer Nature. (i) An illustration of a bifunctional WSe_2_/graphene heterojunction microreactor with the implementation toward water splitting in a neutral medium and (j) polarization curves of the Cr‐doped WSe_2_/graphene heterojunction. Reproduced with permission [185]. Copyright 2022, American Chemical Society. (k) SECCM image of HER photocurrents at −0.96 V vs. Ag/AgCl across the array of anodization defects. Reproduced with permission [186]. Copyright 2020, American Chemical Society.

Ultrafast pump‐probe spectroscopy (UPPS) is an optical‐based technique that uses two laser pulses; a high‐intensity pump pulse to trigger the optical response of the sample, and a weak probe beam is used to investigate the charge carrier dynamics by measuring the changes in transmission mode (see Figure 6a). Pump‐probe techniques employ sampling methods to address limitations in electronic detection, allowing for observing the dynamics of electronic and optical properties on femtosecond or picosecond timescales [169]. The swift progress of ultrafast laser technologies in the last twenty years has propelled advancements in ultrafast pump‐probe spectroscopy. Femtosecond‐scale setups have been reported, with laser systems achieving pulse durations of a few femtoseconds on various electromagnetic spectrums, allowing for a wide range of excitation conditions, ranging from a few picojoules to joules of energy pulsed [170, 171, 172, 173, 174, 175, 176]. Currently, titanium:sapphire (Ti:Al_2_O_3_ or Ti:sapphire) lasers on the market can produce sub‐50 femtoseconds across various power levels [177]. The repetition rate of Ti:sapphire varies from a few hertz to 2 GHz [178]. A higher repetition rate is preferable, as more samplings can be performed for each second acquisition time, improving the experiment's statistical quality. However, it has been known that energy per pulse is inversely dependent on the repetition rate, as higher energy per pulse comes with a lower repetition rate [179].

This method allows for monitoring the ultrafast processes of charge carrier dynamics of nanocatalysts with high temporal resolution, providing insights into electron transfer, trap site recognition, and other critical phenomena. Corp and Schlenker utilised the UPPS technique in the visible and near‐infrared transient absorption (TA) spectroscopy to unveil the process of electron‐transfer cascade in terms of charge separation dynamics for HER [187]. Carbon nitride (g‐C_3_N_4_) has been greatly explored for its promise in driving photocatalytic hydrogen evolution from water due to its excellent mechanical and chemical durability [119, 188]. g‐C_3_N_4_ was produced with thermal condensation of urea, resulting in bulk powder of the material, before two versions of powder were then created; graphitic and exfoliated. Ultrafast TA was used to correlate the excited‐state dynamics of electrons in g‐C_3_N_4_ mixed with chemically exfoliated carbon nitride for photocatalytic activity observation, in which additional oxidative chain termination exists. The electron cascade observed herein includes excitation, exciton dissociation, transfer, and charge separation. After excitation, the charge separation process is carried out: The transferred electrons on exfoliated carbon nitride can participate in subsequent reactions, such as hydrogen evolution, leading to improved photocatalytic activity [189].

Boandoh et al. utilised UPPS to investigate the charge carrier dynamics distribution in different morphological shapes of the atomically thin TMDs, such as molybdenum disulfide (MoS_2_) and tungsten disulfide (WS_2_) monolayers [180]. In this study, four distinct TMD geometries are introduced: Triangular (t‐MoS_2_), curved triangular (c‐MoS_2_), triangular (t‐WS_2_), and hexagonal (h‐WS_2_). Moreover, the transient transmittance changes (ΔT/T) are sensitive to the exact position of the desired measurements, namely, an abrupt change of the decay of photoexcited carriers can be realized at the curved edge and vertices of the TMD. To evaluate the sample homogeneity.

Micro‐Raman mapping of E^1^_2g_ mode of t‐MoS_2_ was carried out. A photosystem structure was used to identify the photo‐electrocatalytic HER mechanism in NiO/Au/Co‐catalyst as depicted in Figure 6b. Here, the transient absorption spectroscopy (TAS) measurement unravels an abrupt change in the decay curvature compared to the NiO/Au and Au catalyst depicted in Figure 6c. Furthermore, sequential ultrafast pump–probe microscopy (UPPM) images shown in Figure 6d correspond to the transient transmittance change ΔT/T span from 0.8 to 40.8 ps of the t‐MoS_2_ flake, suggesting the uniformity of photoexcited carrier dynamics. Based on this scheme, a significant number of defects exist at the edge and the vertex sites, thereby facilitating additional relaxation channels for electrons at the conduction band minimum. In terms of defect‐state recognition, two contrasting polarities arise in which a negative transient transmittance for the case of t‐MoS_2_ and c‐MoS_2_, whereas a positive transient transmittance for t‐WS_2_ and h‐WS_2_ samples after 3–7 ps (defect‐dependent).

Yu et al. also employed femtosecond pump‐probe spectroscopy to characterise the exciton dynamics in the stacked WS_2_ multilayers. This technique provides temporal information on the exciton lifetimes, decay channels, and the competing mechanisms [190]. In this work, the effect of TMD thickness on the dynamic features is attributed to different competition between phonon‐assisted and defect density. For instance, the fast decay process of charge carriers (in this case A excitons; the excitons originate from the top splitting of the VBM caused by strong spin‐orbit coupling and interlayer coupling) at the initial stage is mainly governed by exciton‐exciton annihilation was reduced, thus, the lifetime became increased when the TMD number of layers is thicker. In a different approach, Wang et al. demonstrate three‐pulse (pump‐push‐probe) femtosecond spectroscopy capable to unveil quantitatively an implication of the hot‐phonon bottleneck (HPB) for carrier cooling phenomena in TMDs [191]. The finding indicated that the intrinsic cooling rate can be damped by increasing the number of hot carrier densities. Interestingly, the precise control over the number of cold carriers that could interact with the hot carrier resulted in two proposed relaxation pathways: First is related to the phonon‐assisted due to the extension of the cooling time driven by HPB. Second is related to the carrier‐assisted pathway, where the dominant carrier‐carrier interaction plays a role to push out the cooling process. To correlate the intimate relationship between exciton/trion dynamics and electrocatalytic activity of TMD, Hsiao et al. describe a peculiar carrier relaxation of monolayer MoS_2_ during the HER using in situ time‐resolved microcell‐based liquid microscopy. The corresponding geometric instrumentation used in their study is displayed in Figure 6e. This 2D mapping amplitude ratio is able to determine that a feature of HER arises when trion‐species″ dominates the observation. On the contrary, the “exciton‐dominant” is unveiled via electrolyte gating since the HER process is largely suppressed [182]. Such control on the excitons and trions is heavily influenced by the adsorption/desorption of hydrogen ions or electrolyte ions.

In a further temporal regime, Schumacher et al. devised the message for the unprecedented case of dynamic response in few‐layer CVD‐grown MoSe_2_ by means of attosecond transient absorption spectroscopy (ATAS) technique [183, 192, 193]. A broadband extreme ultraviolet pulse typically evaluates the electronic transition between core and excited states with attoseconds resolution. The overview result is depicted in Figure 6f. This unusual behavior can be exploited using simultaneous element‐and carrier‐specific probing of the excited states in which charge carrier localization is prominently centered at the molybdenum sites. In contrast, the charge dynamics profile probed via the chalcogen atoms displays negligible interactions. This is directly associated to the consequence of the screening effect in *d*‐orbitals. Such a strong localization of electrons within the Mo *d*‐orbitals (final state) is largely understood due to the higher electron‐electron scattering event that can be induced by excitation with the NIR pump pulse.

### 3.2 Nanoscopic Tool for Electrocatalytic HER

In the past decades, the atomic‐scale investigation required a meticulous approach to discriminate the active sites of photocatalytic materials, triggering diverse developments toward the real space visualization of HER [194, 195, 196, 197, 198]. In particular, the SECM technique is increasingly popular in the electrocatalytic fields, because of the combination of spatially resolved voltammetry and simultaneous ion‐conductance measurements. The local correlation of active sites and their kinetics using this technique is indispensable in providing the physical insights into the intrinsic activity of electrocatalysts. To unveil the HER mechanism, the intimate relationship between the local activity of catalysts and their structural origin could be enhanced by optimizing their surface structures and defects [38, 39, 40, 45, 196, 199, 200, 201, 202, 203, 204, 205].

For example, defect engineering in TMDs has been introduced and investigated to enhance HER activity by examining their photoelectrochemical behaviour [184, 186, 199, 206, 207, 208, 209, 210, 211, 212, 213, 214] and their optical responses [215, 216]. This includes the idea of how to understand the kinetic data for HER on these materials and how defects and surface modifications affect their performance. For example, a typical sigmoid shape of a MoS_2_ crystal represents the evolution of proton reduction. Qualitatively, depending on where the measurement point was conducted, normalized linear sweep voltammetry (LSV) can be attributed to distinct materials. This local electrochemical approach is versatile to elucidate the HER profile in the MoS_2_ edge and its corresponding 3D color map based on the substrate generation/tip collection mode shown in Figure 6g,h, respectively. In addition, a key feature is outlined by Wang et al. [184], in which Fast mode was successfully operated, outlining the sharp current distribution on the edge of 2H MoS_2_.In the case of strained MoS_2_ with sulfur vacancies, Hong Li et al. outlined that the HER kinetic data for both unstrained S vacancies (formal potential Ev_0_ = −0.53 V Ag/AgCl, electron‐transfer coefficient αv = 0.4, electron‐transfer rate constant k_v0_ = 2.3 × 10^−4^ cm/s) and strained S_vacancies_ (E_sv0_ = −0.53 V Ag/AgCl, α_sv_ = 0.4, ksv 0 = 1.0 × 10^−3^ cm/s) on the basal plane of MoS_2_ monolayer. This suggests that a strained S vacancy has an electron‐transfer rate 4 times higher than that of the unstrained S vacancy [196]. Despite its advantages, SECM encounters challenges in characterizing the electrochemical activity of catalysts, particularly in complex systems. In particular, the roadmap to enhance the resolution and sensitivity of SECM techniques is an active area of research. In the aspect of theoretical analysis and modeling, these calculation approaches are crucial for understanding the mechanisms of HER. SECM data are often correlated with theoretical models to gain deeper insights into the interfacial descriptors and Tafel constants [205].

In terms of atomic doping engineering, Chiang et al. [185] describe a substantial reduction of the overpotential is found when metallic Cr atoms mediated charge transfer in a 2D ML WSe_2_/graphene heterojunction, as schematically outlined in Figure 6i. To outline the implication of charge doping, the progression of the polarization curve toward lower cell voltage indicates the impact of Cr‐dopant, as depicted in Figure 6j. Scanning electrochemical cell microscopy (SECCM) technique is also capable of defining the distinct activity between several areas of TMD surface, as exemplified in the photocurrent mapping of WSe_2_ nanosheets in Figure 6k.

Recent work by Silva et al. unveiled a contrasting outcome on SECCM image demonstrated for metallic doping of MoS_2_ compared to its respective pristine case. In the spirit to deduce the relationship between the structure and catalytic activity of TMD, the role of hydrogen spillover study introduced by Wang et al. [211] uncovers the implication of strong catalytic enhancement of 1T/2H MoS_2_ heterostructures. A transmission electron microscopy (TEM) image depicted clear separation where an atomic arrangement is clearly defined. Extending the concept of spillover‐mediated HER enhancement to metal–support heterointerface engineering, Shao et al. recently demonstrated a bioinspired ferroelectric HfO_2_‐coupled Ir catalyst (Ir/HfO_2_@C) wherein the catalyst architecture mimics the proton‐management functions of the Mn–O–Ca active centres in photosystem II and the Fe–Fe sites of [FeFe]‐hydrogenases [217]. By exploiting the strong ferroelectric polarisation of HfO_2_, the system establishes pH‐controlled bidirectional hydrogen spillover: under alkaline conditions, H species migrate in the reverse direction from the HfO_2_ support to the Ir interfaces, whereas in acidic media, conventional forward spillover prevails from Ir clusters to the support. This self‐promoting spillover mechanism narrows the intrinsic kinetic gap between the two pH regimes, yielding overpotentials as low as 28 and 18 mV at 10 mA cm^−^^2^ in 1 m KOH and 0.5 m H_2_SO_4_, respectively, with a turnover frequency 20.6‐fold higher than that of benchmark Ir/C. Further technical development of the SECCM method is very critical to spatially resolved electrochemical information on emerging LDMs at the nanoscale.

### 3.3 Advanced Characterization for Thermocatalytic Reaction

To bridge the gap between static catalyst properties and active working states, the emerging field in thermocatalytic process in HER relies heavily on advanced in situ and *operando* characterization techniques [218, 219]. SMR catalysts (for example, Ni‐based) are incredibly prone to oxidation, sintering, or support‐lattice diffusion during dynamic or transient cycles (like startup/shutdown). *Operando* x‐ray absorption spectroscopy (XAS) can be used to monitor when nickel transitions between active metallic phases (Ni^0^) and inactive mixed oxides (Ni^2+^) under steam environments [220]. Reforming reactors could experience steep gradients in concentration and temperature across the catalyst bed. Advanced *operando* profile reactors combine high‐energy XRD with motorized stages and can be used to map phase transformations along the length of the reactor bed in real‐time [221]. In this structural monitoring event, one could estimate a particular site where the nanocatalyst deactivates or phase degradation initiates.

## 4 Low‐Dimensional Materials and Heterogeneous Active Catalysts

Layered LDMs can serve as excellent supports for heterogeneous catalysts, enhancing catalytic activity and selectivity for sustainable HERs, due to their high surface area and unique electronic properties [222]. A key challenge for realizing LDMs‐based electrocatalysts is the controllable and scalable synthesis of high‐quality LDMs. Recent research on non‐noble active materials has emphasized rational nanocatalyst design and the development of engineering strategies such as interfacial engineering, heterostructures, defect engineering, and alloys. The choice of catalyst is critical for the HER, with platinum being the most widely used and most expensive [10, 31, 223]. As outlined in the previous section, extensive direction on MoS_2_ is considered a promising alternative due to its earth abundance and potential for high performance. The development of ideal catalysts requires synergistic optimization of material parameters such as mass active sites, faster mass transfer, well‐defined nanostructures, higher conductivity, and tuned electronic structure. Heterostructure catalysts, made of electrochemically active materials and various functional additives bonded physically or chemically, show significantly enhanced catalytic activity and performance in acidic (0.5 m H_2_SO_4_), neutral (1 m phosphate buffer solution or PBS, seawater), and alkaline (1 m KOH) solutions are briefly summarized in Table 4. To provide an overview of the emerging LDM for HER catalysts, we selectively chosen several breakthrough 2D materials that are composed into heterostructures as the center of discussion.

**TABLE 4:** Heterostructures for HER in acidic, neutral, and alkaline solutions.

			HER Parameters			Water Splitting		Ref.
Catalysts	Heterostructures	Electrolyte	Overpotential @ 10 mA/cm^2^ (mV)	Tafel slope (mV/dec)	Catalytic loading (mg/cm^2^)	Cell voltage (V) @ 10 mA/cm^2^	Durability (h)	
		TMDC‐based heterostructures						
MoS_2_/CoSe_2_	MoS_2_ on the surface of CoSe_2_	0.5 m H_2_SO_4_	68	36	1	1.6	9% decrease @5 000 cycles	[224]
MoS_2_/MoO_2_	MoS_2_/MoO_2_ core‐shell structure	0.5 m H_2_SO_4_	210	129	0.28	1.55	—	[225]
MoS_2_/Fe_3_O_4_	Shell‐core MoS_2_ nanosheets/Fe_3_O_4_ sphere	0.5 m H_2_SO_4_ *iR corrected*	≈ 210	52	0.14	1.40	10% decrease @5 000 cycles	[226]
MoO_3_/MoS_2_	Vertically oriented core‐shell MoO_3_‐MoS_2_ nanowires	0.5 m H_2_SO_4_ *iR corrected*	≈ 250	≈ 55	—	1.40	3 (9% decrease of current density)	[227]
MoSe_2_/NiSe	Epitaxial MoSe_2_/NiSe vertical structure	0.5 m H_2_SO_4_ *iR corrected*	210	56	0.28	1.47 @ 1m KOH	20 @ 1M KOH	[228]
W_x_C@WS_2_	Ravenala leaf‐like W_x_C on WS_2_ nanotubes	0.5 m H_2_SO_4_	146	61	0.30	—	10	[229]
WS_2_/RGO	2D WS_2_ nanosheets on RGO	0.5 m H_2_SO_4_	270	58	0.4	1.23	12	[230]
CoS_2_/Co_3_O_4_	Core‐shell Co_3_O_4_/CoS_2_ nanoneedle arrays on CC	0.5 m H_2_SO_4_	≈ 150	45 Pt/C: 28.2	1.25	1.6	1.5 @ 1 000 cycles	[231]
CoS_2_/CoSe_2_	CoS_2_ nanoparticles dotted on CoSe_2_/DETA nanobelts	0.5 m H_2_SO_4_	80	34	0.28	1.53	500	[232]
Co_3_S_4_/CoP	Porous Co_3_S_4_/CoP nanorods	0.5 m H_2_SO_4_	86	45	0.28	1.53 @ 1M KOH	24 @ 5 000 cycles	[233]
CoP/WS_2_	CoP nanoparticles combined with WS_2_ nanosheets	0.5 m H_2_SO_4_	150	86	0.29	1.78 @ 50 mA/cm^2^	72 @ 1M KOH	[234]
Ni_2_P/MoS_2_	Ni_2_P nanoparticles on MoS_2_ nanoflowers	0.5 m H_2_SO_4_ *iR corrected*	≈ 200	76 Pt/C: 55	—	1.23	8	[235]
Cu_2‐x_S/Ru	Cactus‐like hollow Cu_2‐x_S/Ru nanoplates	1 m H_2_SO_4_	82	48	0.23	1.47 @ 0.1 m HClO_4_	12	[236]
Ag_2_S/Ag	Ag_2_S/Ag nanowires	0.5 m H_2_SO_4_	199	102 Pt/C: 37	1.06	—	—	[237]
MoS_2_	5.2 wt.% Rh‐MoS_2_	0.5 m H_2_SO_4_	47 Pt/C: 26	24 Pt/C: 30				[158]
Ni‐graphene‐CNTs‐Ni_2_P‐CuP_2_	Ni‐Foam‐Graphene‐Carbon Nanotubes	0.5 m H_2_SO_4_ *iR corrected*	12	41 Pt/C: 38 at 1 m KOH				[161]
2D porous MoP/Mo_2_N		0.5 m H_2_SO_4_	89	53				[238]
1T‐WS_2_|P‐5		0.5 m H_2_SO_4_ *iR corrected*	125 20 wt.% Pt/C: 69.84	73.73				[239]
2D/2D/1D TiVCT*_x_*/MoS_2_‐CNT		0.5 m H_2_SO_4_ *85% iR corrected*	180	79				[163]
Mo single atoms@1T‐CrS_2_		0.5 m H_2_SO_4_	97 (Pt: 42)	∼52 (Pt: 31)				[164]
		Hydroxide / Mixed‐Phase Heterostructures						
TiO_2_ NDs/Co nanotubes	Co nanotubes decorated with TiO_2_ nanodots	1 m KOH	106	62	0.75	—	100	[240]
MoSe_2_/Ni_0.85_Se	3D MoSe_2_/Ni_0.85_Se nanowire network	1 m KOH	117 Pt/C: 42	66 Pt/C: 41.3	6.48	1.42	40	[241, 242]
1T‐MoS_2_/ Ni_2+δ_O_δ_(OH)_2−δ_	Nickel hydr(oxy)oxide nanoparticles on 1T MoS_2_ nanosheets	1 m KOH	73 Pt:32	75 Pt 41	0.4	1.60	30	[46]
RuCo/NC	RuCo nanoalloys encapsulated in nitrogen‐doped graphene	1 m KOH	28 Pt:40	31 Pt:31	0.28	1.52	24 @ 2 000 cycles	[243]
RuO_2_/NiO	RuO_2_/NiO nanorods arrays on NF	6 m KOH	100 (@ 100 mA cm^−2^) Pt‐Dy:400 @ 100 mA cm^−2^	38.5 Pt‐Dy: 147	0.14	1.43	20	[244]
0.2CoSe_2_/MoSe_2_	Cubic phase CoSe_2_ quantum dots decorated on MoSe_2_ surface	1 m KOH *95% iR correction*	218	76	0.20	1.52	12	[245]
Ni‐P/MoS_x_	Electrodeposited hybrid Ni‐P/MoS_x_ film	1 m KOH *iR correction*	140	64	—	1.52 @ 100 mA/cm^2^	10	[246]
NiO/Ni‐CNT	Nanoscale NiO/Ni heterostructures formed on CNT sidewalls	1 m KOH	20 (@ 20 mA cm^−2^) (Pt/C:50 (@ 100 mA cm^−2^)	51 (Pt 41)	0.28	1.5 @ 20 mA/cm^2^	24	[42]
Ni(OH)_2_/CuS	Mesoporous nano‐shelled amorphous Ni(OH)_2_/crystalline CuS hollow spheres	1 m KOH *iR correction*	95 Pt: 42	104 Pt:48	0.29	1.5	30	[247]
Ag‐Ni	Core/shell Ag/Ni nanowires	0.1 m KOH	197	84	0.12	—	3	[248]
Fe_2_P/Co_2_N		1 m KOH	29	40.2		1.561	120	[160]
2D porous MoP/Mo_2_N		1 m KOH	89 Pt/C:40	78 Pt/C:54		1.54	48	[238]
Ni‐graphene‐CNTs‐Ni_2_P‐CuP_2_	Ni‐Foam‐Graphene‐Carbon Nanotubes	1 m KOH *iR correction*	32			1.45	40	[161]
Ni_2_P/NiMoP		1 m KOH *iR correction*	22 Pt/C:16	34.5 Pt/C:29.5		1.35 (urea‐assisted)	80	[159]
He_1e13_‐MoSe_2_		1 m KOH *iR correction*	90	49				[249]
1T‐WS_2_|P‐5		1 m KOH *iR correction*	190 Pt/C:90	92.11 Pt/C:63.24				[239]
		Neutral pH (PBS) electrolytes						
Cu_0.08_Co_0.92_P	nanosheet arrays on carbon paper	1 m PBS	81	83.5		1.72	60	[47]
2D porous MoP/Mo_2_N		1 m PBS	91 Pt/C:37	51 Pt/C:34		1.54 in 1 m KOH	48	[238]
		Other electrolytes						
NiCoN|Ni*_x_* P|NiCoN		Seawater	165	139.2 Pt/C:233.6		1.81	24 (increase of ∼50 mV)	[162]

### 4.1 Layered Double Hydroxides

**FIGURE 7:**
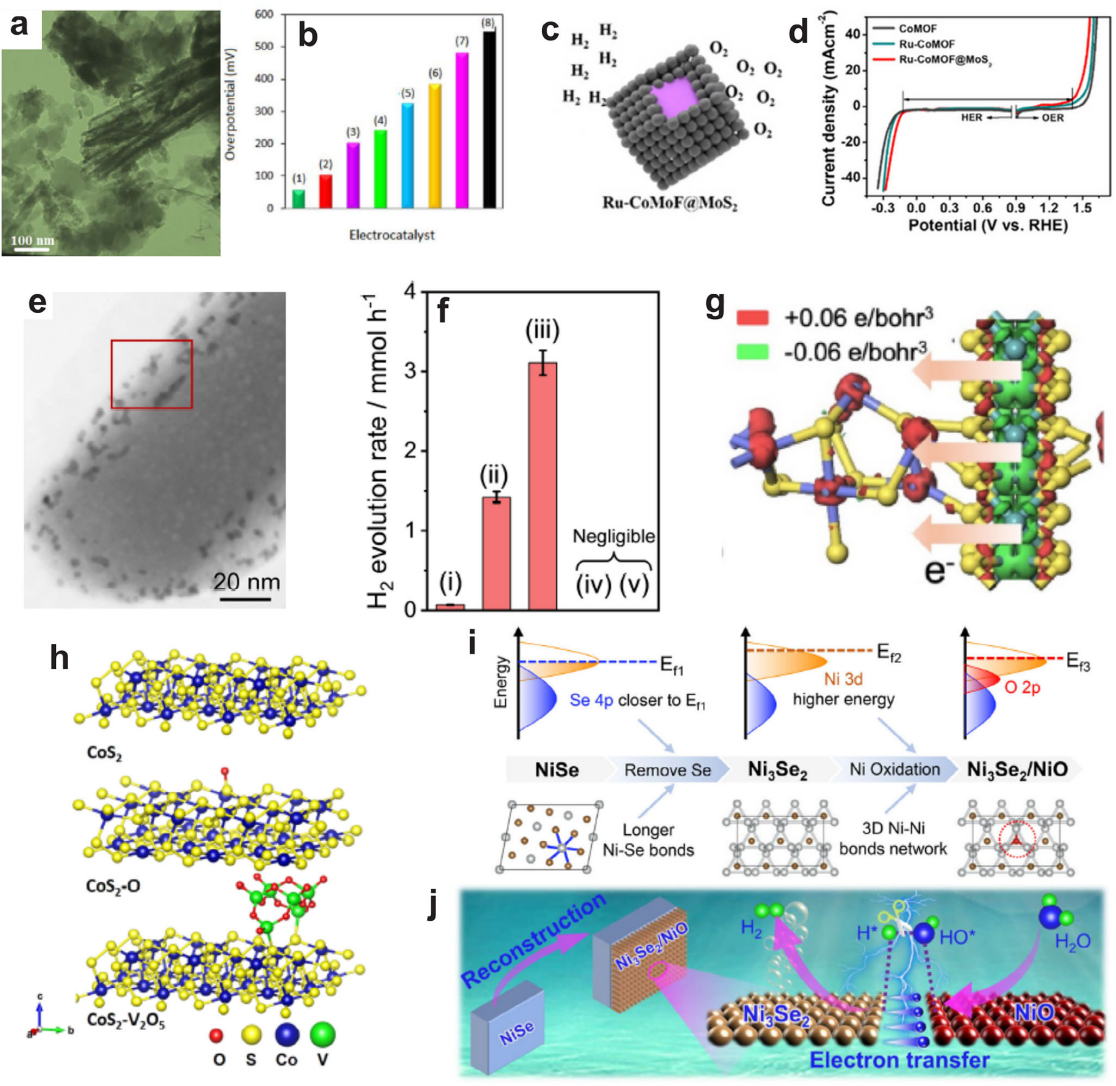
Several highlights of emergent LDMs converted into a heterostructure for H_2_ generation. (a) TEM image of NiMn‐LDH/CuCo_2_S_4_/rGO nanocomposite and (b) overpotential values measured at 10 mA cm^−2^ current density with the pristine components. Reproduced with permission [251]. Copyright 2026, The Royal Society of Chemistry. (c) Representation of disrupted Ru‐CoMOF@MoS_2_ and (d) reversible current profile of composite. Reproduced with permission [254]. Copyright 2022, Wiley‐VCH GmbH. (e) Bright field‐STEM image of Cr_2_O_3_/Pt/IrO_2_/ Gd_2_Ti_2_O_5_S_2_ composites and (f) H_2_ evolution of composites compared to the pristine counterparts. Reproduced with permission [255]. Copyright 2022, Wiley‐VCH GmbH. (g) Charge density difference plot of 2H‐MoS_2_/CoS_2_ heterostructure suggesting the progression of charge carriers. Reproduced with permission [256]. Copyright 2026, Elsevier B.V. (h) Calculated crystal structure of CoS_2_ protected from oxidation by V_2_O_5_. Reproduced with permission [257]. Copyright 2026, The Royal Society of Chemistry. (i) NiSe to Ni_3_Se_2_/NiO and (j) reconstruction profiles of dual‐site HER. Reproduced with permission [258]. Copyright 2026, Wiley‐VCH GmbH.

The incorporation strategy of layered double hydroxides (LDHs) with other conductive materials or substrates, bolstered by strong interfacial interactions and defect engineering, could enhance their electrical conductivity, stability, and catalytic activity. Moradi et al. construct of hierarchical heterostructures of MoS_2_@NiFeCo‐Mo(doped)‐LDH with good performance toward HER and accompanied by a low IR‐corrected overpotential of 61 mV at 10 mA cm^−2^ under light illumination, which is better in comparison to the benchmark Pt/C (73 mV) [250]. Driven by the multisite performance, a trifunctional noble‐metal‐free based on NiMn‐LDH/CuCo_2_S_4_/rGO nanocomposite (TEM image shown in Figure 7a) proposed by Abazari et al., offers unique structural characteristics with a fast charge transfer profile. The composite achieves a low overpotential of 101 mV at 10 mA cm^−2^ current density and a low Tafel slope of 190 mV dec^−1^ (Figure 7b). This strategy lies in controlled phase interfaces, enhanced electrical conductivity, and fast electron transport [251]. Z. Guan et al. utilize the concept of effective interactions between nanoclusters with nanosheets to produce more active sites compared to the pristine materials. FeCoCe LDH@NiSe heterostructures in a symmetric electrolyzer configuration deliver 10 mA cm^−^^2^ at 1.8 V with negligible activity decay during 45 h continuous testing under alkaline operation. This is governed by the strong interfacial effect between FeCoCe LDH and NiSe, reducing the transmission pathway across the heterostructures and thereby increasing the rate of electron transfer [252]. In the S‐scheme heterojunction of sulfur‐vacancy‐modified Mn_0.3_Cd_0.7_S (MCS‐s) and NiCr‐layered double hydroxide (NCL), the interplay between 1D MCS‐s and 2D NCL demonstrates HER efficient of 42.78 mmol g^−1^ h^−1^, exceeding the performances of pristine MCS‐s and NCL by 1.87 and 85.6 times, respectively. Here, sulfur vacancies alter the band structure and promote an internal electric field at the heterointerfaces that shorten charge carrier diffusion pathway [253].

### 4.2 Metal Organic Frameworks

A strong coupling between metal organic frameworks (MOFs) and LDMs can be materialized due to an atomically ordered and porous network of the MOF structures that meets the low dimensionality order of conducting layered LDM structures [259, 260, 261]. By performing a confinement of LDM within MOF structures, Qiao et al. infer that MOF networks prohibited the stacking of MoS_2_. This allows to promote charge transport and enhances active sites. The MOF/MoS_2_ composite photocatalyst is reported with the maximum hydrogen production rate of 626.3 µmol g^−1^ h^−1^ [262]. In terms of rational design, MOFs can be maximized by tailoring the interactions at the heterointerface regime. Banti et al. reported that the progression channel for electron transfer in Ru‐CoMOF@MoS_2_ heterostructures (Figure 7c) can produce a low overpotential of 240 mV, an enhanced Tafel slope of 87.9 mV dec^−1^, and excellent stability of 60 h. The versatility of the heterostucture is outlined in the total polarization shown in Figure 7d. This is possible since the Co site maximizes the evolution of hydrogen intermediate energy for adsorption and enhances HER, benefitting heterointerface as a channel for electron transfer and promoting reactions at the solid‐liquid interface [254]. In the case of bi‐metallic MXene (Mo_2_TiC_2_T_x_)@NiCo‐MOF composite (B‐MNC), Jalalah et al. used a green hydrothermal method to synthesize the B‐MNC electrode with a Tafel slope of 45 mV dec^−1^ and a low overpotential of 40.42 mV [263]. A standing issue in this composite is related to the long‐term stability, for example, MXene‐MOF hybrids reported by Chellasamy et al. highlighted a drawback in which MXene structure integrity is susceptible in acidic media [264].

### 4.3 Non‐Stoichiometric Chalcogenides and Oxychalcogenides

Recent breakthroughs in stoichiometric and non‐stoichiometric semiconductor chalcogenides, covering metal‐rich sulfides, selenides, tellurides, and pentlandites, have emerged as high‐performance electrocatalysts for the HER development [265, 266, 267, 268, 269, 270, 271, 272]. By deliberately introducing defects, vacancies, or excess metal atoms, these materials surpass the limitations of ideal stoichiometry, enhancing conductivity and optimizing the Gibbs free energy for hydrogen adsorption (ΔG_H_). In the advancement of nanostructures, yolk@shell configuration successfully competes with the conventional electrocatalysts [273, 274, 275]. For instance, Au@NiS_x_ nanostructures showed its catalytic promise owing to the conductive NiS_x_ component and active surface high‐valence Ni^3+^ species [273]. Chun‐Wen Tsao et al. reported that the generated plasmonic feature of Au@Cu_7_S_4_ exhibited a promising quantum yield of 7.3% at 2 200 nm without additional co‐catalysts [274]. A van der Waals–covalent bonding interface composed of a MoS_2_‐to‐Mo_5_N_6_ heterostructure is introduced by Xinying Luo et al. to exploit structural distortion control on the HER activities [265].

Oxychalcogenide materials (containing both oxygen and chalcogenide anions) are emerging as highly efficient, stable catalysts for HER, specifically designed for photo‐ and electrocatalytic water splitting [276, 277, 278, 279]. These mixed‐anion materials combine the stability of oxides with the high electronic conductivity of chalcogenides, often exhibiting high‐entropy structures with multiple active sites that enable low overpotentials (52–57 mV) and durability in both acidic and alkaline environments. These exciting 2D ultrathin sheets have shown their versatile photocatalysts with promising gas evolution rates. For example, Gd_2_Ti_2_O_5_S_2_ with atomically ordered surfaces with Pt, Ir, and Cr_2_O_3_ serve as cocatalysts, and the corresponding STEM image shown in Figure 7e exhibited the H_2_ rate of 3 000 µmol h^−1^ (Figure 7f) [255], and Y_2_Ti_2_O_5_S_2_ nanosheets yield the H_2_ rate of 141 µmol h^−1^ [280]. In terms of hydrogen spillover, high‐entropy oxychalcogenides (HEOC) plays substantial contribution toward multiple active sites. Kim et al. reported HEOC with low overpotentials of 52 (acidic) and 57 mV (alkaline) to obtain a current density of 10 mA cm^−2^ [279]. The intricate features of rational design in this class of materials are heavily influenced by the cation choice, polarity aspect, band structure modulation, and connectivity.

### 4.4 Transition Metal Selenides, Phosphides, Sulphides

The transition from noble‐metal catalysts to earth‐abundant alternatives has catalyzed an intense research initiative in HER electrocatalysis, positioning transition metal chalcogenides and pnictides, particularly sulfides, selenides, and phosphides as leading candidates [281, 282, 283]. Over the past five years, these materials have evolved from simple binary compounds to highly engineered nanostructures with tunable electronic states, abundant active sites, and optimized hydrogen adsorption energetics. Their intrinsic advantages, such as metallic or semi‐metallic conductivity in phosphides, rich defect chemistry in sulfides, and flexible electronic structures in selenides, enable accelerated charge transfer and favorable reaction kinetics, addressing the limitations of high‐cost Pt‐based systems.

In the latest development of transition metal selenides‐based in all‐around pH catalyst, Zhen et al. propose a ReSe_2_/NiSe heterostructure displaying excellent HER performance in a wide pH range [284]. Their finding suggests that low overpotentials of 101, 111, and 154 mV can be reached with the current density of 10 mA cm^−2^ in acidic, alkaline, and neutral conditions, respectively. At a high current density of 100 mA cm^−2^, the activity in both acid and alkali, even surpassing that of commercial Pt/C is somewhat driven by the micro‐rod array configuration of ReSe_2_/NiSe, which promotes the release of hydrogen bubbles during the HER process, thus accelerating the reaction kinetics. In another case, the trimetallic system of Zn_0.1_Co_0.3_Ni_0.6_Se_2_ NPs is reported with excellent electrocatalytic activity toward HER with an overpotential of 121 mV at 10 mA cm^–^^2^ and a relatively low Tafel slope of 35.3 mV dec^–^^1^ [285]. The electrocatalyst also displays good long‐term stability for a duration of 12 h at 10 mA cm^–^^2^ in 0.5 m H_2_SO_4_. The proposed discussion unveils that the combination of the trimetals in optimal ratio may promote good electronic conductivity and active surface area. In cationic engineering, Sagayaraj et al. reported the incorporation of Cu in nickel selenide catalyst to improve HER activity in alkaline water. This introduction allows to deliver a current density of −10 mA cm^−2^ with a lower overpotential of only 45 mV, overcoming Pt at all potentials under identical conditions [286].

The heterojunction, interfacial, and dopant engineering remains as interesting approach to tune the transition metal sulphide‐based catalysts. Yang et al. outline a hierarchical ReSe_2_/ZnCdS S‐scheme heterojunction capable to produce hydrogen evolution rate of 13.96 mmol g^−1^ h^−1^ under visible light irradiation, this is higher than pristine ZnCdS (2.36 mmol g^−1^ h^−1^) [287]. This photocatalytic performance is mainly governed by the enhanced light‐harvesting efficiency in the disordered nanoflower‐like ReSe_2_ and the efficient separation of photogenerated electrons and holes in the S‐scheme heterojunction. Ni_2.3_CoP/Ni_3_S_2_@NF heterojunction featuring a peculiar shape of “ball‐like surface with rough edges and a porous structure” is introduced by Mahmood et al., with a lower overpotential of 23.1 mV at a current density of 10 mA cm^−2^ [288]. Furthermore, Xia et al. reported that CoSe_2_/Mo_2_C heterostructure encapsulated within 2D carbon nanosheets yields strong interfacial electron interactions and an efficient d‐band center for the active sites of Co and Mo, enhancing the bifunctional water splitting [289]. A synergistic edge–interface strategy of CoS_2_/MoS_2_ heterostructures was employed by Zhao et al. to deliver an HER current density of 10 mA cm^−2^ at an overpotential of 84.6 mV with a Tafel slope of 49.22 mV dec^−1^ and a durability of 50 h of stable operation at an industrial‐level current density of 300 mA cm^−2^ in a two‐electrode electrolyzer. To shed some light on the governing mechanism, a charge density difference plot is presented in Figure 7g [256]. Zhang et al. propose Re_3_P_4_/ReS_2_ heterostructure featuring an Ohmic contact interface and bidirectional electron flow, which acts as an efficient pH‐universal catalyst for HER with overpotentials of 40–94 mV and, in an AEMWE, maintains stable operation for 500 h at an industrial current density of 500 mA cm^−^^2^ [290]. In terms of surface oxidation protection strategy, Jie Wu et al. describe that by introduce V_2_O_5_ nanoclusters onto CoS_2_ could potentially afford promising HER performance, DFT calculations were performed to predict the lowest surface oxidation degree, as shown in Figure 7h.

Ding et al. reveal an unprecedented phase transition of NiSe into a Ni_3_Se_2_/NiO heterostructure during HER. Figure 7i shows the proposed heterostructure [258]. It turns out that the active species experience reconstruction, where the extension of Ni‐Se bonds and a Se 4*p* band position closer to the Fermi level promote the NiSe to Ni_3_Se_2_ transformation. Meanwhile, in Ni_3_Se_2_, a 3D Ni‐Ni bond network and high‐energy Ni 3*d* bands facilitate surface oxidation to NiO. Ni_3_Se_2_/NiO interface yields water dissociation via OH* linkage on Ni sites of NiO and H* on Se sites of Ni_3_Se_2_. Figure 7j outlines the reconstruction of NiSe, while optimized Se site electronic structure promotes H_2_ generation. The reported overpotential was achieved at 80 mV at a current density of 10 mA cm^−2^.

Multi‐heterostructured bifunctional catalysts of transition metal phosphide are greatly influenced by their composition and morphological aspect. In the case of a cobalt–nickel–iron–phosphorus–sulfur composite, Yang et al. formulate 2D/3D hierarchical structure with promising HER performances at 63 and 235 mV at current densities of 10 and 100 mA cm^−2^, respectively [291]. To emulate the impact of compositional engineering in a high‐entropy metal phosphide, Wang et al. hypothesized that metal–P bonds can be used to reduce metal–metal interaction and consequently decrease the free energy of hydrogen adsorption, contributing the enhanced catalytic performance with an overpotential of 26 mV at 10 mA·cm^−2^ and Tafel slope of 50.9 mV·dec^−1^ [292]. Qian et al. exploit Re_2_P/ReS_2_ heterostructures as electrocatalysts with abundant reaction active sites and strong electronic interaction between ReS_2_ and Re_2_P, thus enhancing the catalytic activity with overpotentials of 69 and 57 mV at 10 mA cm^−2^ for HER in alkaline and acidic media, respectively [293]. Recent work by Yuan et al. exemplified the importance of a multiphase heterogeneous structure to facilitate abundant interfaces with more active sites, where for NiMnP/Ni_2_P/MnP_4_/NF composite, a current density of 10 mA cm^−2^ can be achieved at 57 and 74 mV for HER overpotentials, close to the Pt/C catalyst of 31 and 57 mV, in acidic and basic electrolytes, respectively [294].

Recent demonstrations on interfacial engineering, defect modulation, and heteroatom doping can significantly lower overpotentials and enhance long‐term durability, even under industrial current densities in diverse classes of transition metal series. Moreover, the importance of edge‐site activation, basal‐plane engineering, and dynamic surface reconstruction during HER becomes a main ingredient to collectively develop toward a paradigm shift into multicomponent, interface‐rich nanocatalysts, bringing non‐noble catalysts based on transition metals increasingly closer to practical hydrogen production technologies.

Gao et al. reported a MoS_2_/CoSe_2_ heterostructure catalyst by depositing MoS_2_ on the surface of CoSe_2,_ which exhibited an excellent HER performance in 0.5 m H_2_SO_4_ with an overpotential of 68 mV at 10 mA/cm^2^, a Tafel slope of 36 mV/dec, and a catalytic loading of 1 mg/cm^2^ [295]. Chen et al. synthesized RuCo nanoalloys encapsulated in nitrogen‐doped graphene, and the RuCo/NC heterostructure delivered 10 mA/cm^2^ at a very low overpotential of merely 28 mV in 1 m KOH [243]. From the HER catalytic activity point of view, the components in a heterostructure could be either active or inactive and exhibit higher HER activity than the single counterpart due to a variety of advantages offered by a heterostructured configuration. However, creating heterostructured HER catalysts is a great way to increase the number of active sites. These catalysts have fine nanostructures with many exposed edges, providing ample adsorption sites for HER intermediates. Adding macroscopic substrates like nickel foam or carbon cloth can further increase active sites, enhance electrical conductivity, and enable fast mass diffusion, reducing overpotential at high current densities [224, 296, 297]. Furthermore, constructing well‐defined nanostructures, such as core‐shell structures, nanosheets, nanowires, nanoneedles, nanorods, nanobelts, nanoflowers, nanoplates, and nanodots, can improve the durability of catalysts by protecting unstable species with stable ones.

The difference in electronegativity between components in heterostructure catalysts can induce electron transfer between them, and redistribution will regulate the electronic structures or the band structures of the components, which is critical to the superior HER activity. Finally, the so‐called synergistic effect also contributes significantly to enhanced HER kinetics of the heterostructures [233]. For example, the MoS_2_/CoSe_2_ nanobelts heterostructure with increased catalytic sites is likely due to the electrocatalytic synergistic effects between hydrogen evolution‐active MoS_2_ and CoSe_2_ materials [295]. CoSe_2_ not only couples with MoS_2_ to enhance HER activity but also supports the growth of MoS_2_, forming more active sites. Additionally, the anchored MoS_2_ further boosts the HER‐active sites of CoSe_2_, collectively leading to high HER performance. In addition, a recent interest to utilize synergistic interactions of trimetallic heterostructures is introduced [298]. A synergistic co‐confinement strategy using dual‐heteroatom co‐confinement within a single‐phase host represents a notable advance in the design of MoS_2_‐based heterostructure electrocatalysts. An overpotential of 382 mV at an industrially relevant current density of 1 000 mA cm^−^^2^ in acidic media, substantially lower than the 671 mV required for commercial Pt/C under identical conditions, and maintained stable operation for over 360 continuous hours, establishing dual heteroatom co‐confinement as a practical route to high‐current‐density HER performance [299]. Recent interplay between geometric and electronic manipulation of LDM is proposed, for example, the hierarchical structure consisted of a tri‐component electrocatalyst, MoS_2_@CoS_2_/MXene. that achieved a catalytic performance (current density) of 166.3 mV (10 mA cm^−2^) in acidic conditions. This can be accomplished via vertically anchored MoS_2_ nanosheets onto a CoS_2_ framework, followed by electrostatically self‐assembled of conductive Ti_3_C_2_T_x_ Mxene [300].

## 5 Artificial Intelligence and Machine‐Learning‐Driven Discovery of Non‐Noble Metal Catalysts

Machine learning (ML) has emerged as a transformative paradigm for the design and discovery of electrocatalysts and photocatalysts by enabling the rapid exploration of vast materials chemistry spaces that exceed the capacity of traditional experimental or first‐principles methods. The integration of ML into this field involves a systematic workflow of data acquisition, the development of mathematical descriptors for materials featurization, and the training of predictive models to identify high‐performance candidates. By leveraging these data‐driven algorithms, researchers can efficiently screen novel materials and elucidate complex catalytic mechanisms, thereby accelerating the development of sustainable technologies for clean fuel generation and environmental remediation [301, 302]. The integration of ML into 2D materials research has further revolutionized electrocatalyst design by employing the knowledge discovery in databases (KDD) approach to systematically mine historical data for the acceleration of material screening and performance optimization. A notable application within this framework includes using ML to predict the HER activity of various 2D TMDs, allowing for the identification of optimal surface configurations and doping strategies without the need for exhaustive trial‐and‐error experimentation [303].

The integration of artificial intelligence into thermochemical processes like SESMR provides a powerful solution for upscaling by using data‐driven soft sensors to estimate unmeasurable reactor variables in real time. These ML models, such as random forests and artificial neural networks, leverage input process features like the sorbent‐to‐carbon ratio and temperature to accurately predict gas concentrations and material performance where physical measurement is limited [304]. In another study, the proposed universal microkinetic‐machine learning (mkml) method successfully identified 48 promising bimetallic candidates from a database of over 5 000 materials, significantly accelerating the discovery of efficient and cost‐effective catalysts for steam methane reforming [305]. The ML framework also successfully correlated computational transition‐state energies with experimental data to pinpoint the specific C─C bond‐scission reactions that determine the selectivity of ethanol reforming. By applying this model to screen bimetallic surfaces, researchers identified promising catalyst candidates such as Cr‐Pt‐Pt(111) that optimize the balance between hydrogen production and carbon‐oxygen bond breaking [306]. The application of ML extends beyond material discovery to the holistic optimization of production systems, where advanced algorithms enable real‐time predictive maintenance and precise control over varying operational parameters [307]. By leveraging these data‐driven insights, the efficiency of steam reforming and electrolysis processes is significantly enhanced, ultimately reducing costs and accelerating the transition toward a sustainable green hydrogen economy.

Beyond individual material classes, the integration of artificial intelligence and ML is fundamentally reshaping the entire hydrogen technology landscape by providing advanced predictive simulations and multi‐objective optimization that accelerate the transition from lab‐scale catalyst discovery to cost‐effective, industrial‐scale green hydrogen production [308]. Furthermore, deep learning architectures such as crystal graph convolutional neural networks (CGCNNs) have been successfully employed to screen thousands of candidates from 2D material databases, accurately identifying 38 high‐performance catalysts by predicting adsorption energies across all potential active sites with a speed significantly surpassing traditional density‐functional‐theory calculations [309]. Additionally, ML has been applied to transition‐metal‐based catalysts to identify key descriptors like the d‐band center and coordination numbers, which has enabled the high‐throughput discovery of efficient non‐precious metal electrocatalysts by predicting their Gibbs free energy of hydrogen adsorption with high precision [310]. To conclude the development of high‐efficiency screening tools, recent work has demonstrated an extremely randomized trees (ERT) model that utilizes a minimized set of only ten key features, including a novel energy‐related descriptor, to predict hydrogen evolution reaction activity across multi‐type catalysts with R^2^ = 0.922, achieving a prediction speed 200 000 times faster than traditional density functional theory methods [311].d

Ultimately, the adoption of advanced models like ERT demonstrates that high‐throughput screening can be achieved with a minimal set of key energy‐related descriptors while maintaining exceptional predictive accuracy. This shift toward feature‐minimized ML not only provides a high‐speed alternative to conventional computational methods but also establishes a scalable framework for the rapid discovery of multi‐type catalysts essential for green hydrogen production [311]. Furthermore, the integration of generative AI with automated experimental platforms has established a “closed‐loop” autonomous discovery system, which significantly reduces human intervention while optimizing the multiscale design of electrodes from the atomic to the system level [312]. This shift toward self‐optimizing laboratories represents the current frontier in catalysis research, providing a robust and scalable framework to overcome the remaining efficiency barriers in green hydrogen production.

## 6 Industrial Application Challenges and Prospects

The transition from laboratory‐scale breakthroughs to industrial application of non‐noble metal catalysts toward green H_2_ production in recent years has focused on bridging the performance‐durability gap concept. While noble metals like Pt set the benchmark, emerging research has identified that interfacial electronic modulation in heterostructures can match these kinetic limitations at a fraction of the cost. Hence, the focus has shifted from high‐precision laboratory methods such as chemical vapour deposition (CVD) or atomic layer deposition (ALD) to top–down and high‐throughput bottom–up techniques. These methods prioritize scalability, cost‐effectiveness, and the ability to produce catalysts in kilogram quantities while maintaining the synergistic interfaces necessary for rapid H_2_ production. In thermocatalytic H_2_ production, excellent performance is obtained under optimized laboratory conditions with dilute feeds and short testing periods, whereas industrial reactors require continuous operation for thousands of hours, scalable synthesis routes, high mechanical robustness, and resistance to thermal cycling. The mismatch between academic metrics (e.g., peak activity and atom efficiency) and industrial requirements such as regeneration capability, manufacturability, and techno‐economic viability become the challenges in commercialization and adoption.

In the past two years, two subjects on the performance gap for industry application are the following. First, the loading mass transport at high current, here, we note that the laboratory metrics for HER tested at 10 mA/cm^2^ do not translate to industrial needs (500–1 000 mA/cm^2^). Second, long‐term robustness, while most reported studies indicated 50–100 h of stability is the commonly reported operating time, however, industrial benchmarks require about >5 000 h. This in turn, remains as the biggest challenge in non‐noble metal catalyst research. Here we also outline the three typical challenges: large‐scale synthesis, long‐term stability, and cost control. The batch‐to‐batch inconsistency in hydrothermal and solvothermal methods used to grow nanocatalysts led to nanoparticle aggregation that creates high rejection rates and low throughput. Long‐term stability is related to catalyst leaching or dealloying under high current densities (>1 A cm^2^) and bubble‐induced mechanical stress. This, in turn, creates an economically short stack of lifespan, in which the design of experiments targets >5 000 h, while many non‐noble catalysts are failing at <200 h. In terms of cost control, the main problem is due to an expensive precursor and energy‐intensive heating protocols. For example, 500g‐scale batches of Ni‐based heterostructure were successfully produced in a single annealing step [313] and a bifunctional Ir/ZnNNi_3_ heterostructure with operational stability up to 100 h at 500 mA cm^−2^ [314].

## 7 Concluding Remarks and Future Perspectives

**FIGURE 8:**
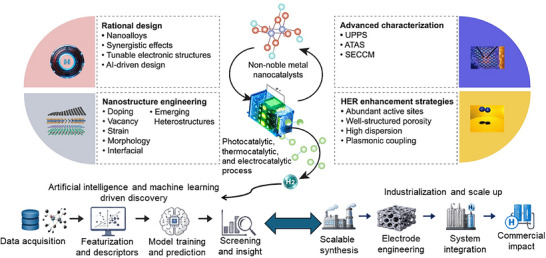
Emergent aspects in catalytic performance enhancement using non‐noble metal nanocatalysts.

Recent research on non‐noble active materials has emphasized rational nanocatalyst design and advancement for efficient HER. To this end, we summarize several interrelated strategies that are expected to play important contributions toward the high performance of nanocatalysts, schematically outlined in Figure 8. Careful material selection enhances light‐to‐electricity conversion in photo‐/electro‐/thermocatalysts and advances understanding of catalytic mechanisms, such as H* intermediate formation and surface effects. Each non‐noble nanocatalyst type presents distinct advantages, limitations, and design strategies for future development. By outlining an overview of H_2_ production, H_2_ selectivity, coking rate, and space velocity, our desire is to provide a generic ingredient how to boost toward highly efficient and stable active nanomaterials. Recent updates on other types of materials, such as perovskites [315, 316, 317], zeolitic imidazolate framework metal organic framework composites [318, 319, 320], transition metal carbo‐chalcogenides [321], and transition metal oxides [322] devise future promise of the relevant fields. In addition, theoretical work to incorporate single‐atom catalysts onto desired 2D materials [4, 323] carry a good leverage in the diverse class of material libraries. We therefore believe that data‐driven efforts in combination with ML [301] would substantially advanced the materials discovery in nanocatalyst exploration. Emerging studies have developed that several ML‐driven predictions, such as extremely randomized trees, gradient boosting, and random forest regression, evaluating the experimental synthesis and validation of novel non‐noble alloys and heterostructures with promising HER performance. By integrating physical knowledge (like Brønsted–Evans–Polanyi relations), a clarification of which material properties drive high HER performance. These constructive approaches improve both transparency and trust in ML‐aided discoveries, providing actionable insights into active‐site design and catalytic optimization. For example, ML‐predicted non‐noble metal photocatalysts such as Fe/g‐C_3_N_4_ composites have demonstrated strong experimental HER performance. Ensemble regressors accurately predicted the hydrogen evolution rate with R^2^ values up to 0.9895, confirming high correspondence between predicted and experimental results [302].

Natural gas reforming, such as SMR is a mature and common commercial H_2_ production technology. While metallic nickel catalysts have demonstrated effectiveness in this H_2_ production process, their stability and performance at low temperatures require further improvement. Moreover, a significant amount of CO_2_ emission released from this reaction has prompted to the extensive efforts of the use of CO_2_ capture and storage technologies. Alternative approaches such as SESMR, which integrates in situ CO_2_ absorption, have emerged to mitigate environmental concerns. On one hand, in this blue H_2_ production, continued research will focus on the search for heterogeneous non‐noble metal catalysts along with lower costs but still high catalytic activity and long‐term thermal stability. On the other hand, developing more environmentally H_2_ production routes will remain a crucial topic. Research and development strategies have been directed to a sustainable transition toward green H_2_ production harnessing all the renewable resources, such as steam reforming using biofuel feedstocks, solar‐driven water splitting, and/or renewable energy‐powered electrolysis.

Finding high‐performance non‐noble metal‐based nanocatalysts for the HER is critical to advancing a sustainable hydrogen economy. As being well known, high surface area, a sufficient amount of active sites, and well‐structured porosity are the primary requirements for catalysts. In steam reforming, a high dispersion of catalysts on the support has an impact on the catalytic activity. Here, catalyst supports with high thermal and hydrothermal stability are desired to ensure that their structure and active sites remain largely unchanged during long‐term working conditions. Heterogeneous nanocatalysts with the complexity of material composition and surface structures offer flexibility in controlling the reactivity and selectivity. Alloying two or more non‐noble metals, the (edge/corner) position of active sites, and surface exposition of reaction‐favoring lattice planes may be further exploited to further enhance the catalytic performance. Current developments have stimulated the use of high‐entropy alloys and medium‐entropy alloys consisting entirely of non‐noble metals. The intention is to facilitate a scalable synthesis that potentially produces low overpotentials and high mass activity at ultra‐low cost. In this scenario, the electronic modulation through optimal alloying improves intrinsic HER activity by reducing hydrogen adsorption free energy. In addition, a combination of first‐row transition metals (such as Ni, Co, Cu, Mn, Fe, Mo) in various nanostructures operates synergistically to drive the catalytic efficiency, as noted in overpotentials, Tafel slopes, and cycling durability. Several operational conditions other than solution composition significantly impact the effectiveness of catalysts in water splitting kinetics and the resulting hydrogen production, such as temperature and/or pressure [324, 325]. Moreover, the supplementary function of catalysts may be added to reduce unwanted reactions potentially causing catalyst deactivation, such as coke deposition in SMR and ESR. The use of materials with high oxygen storage capacity and the ability to release oxygen may be further explored to mitigate the parasitic coke deposition, considering transition and rare‐earth metals with a variety of coordination numbers.

In photocatalytic H_2_ production, the catalysts should have good semiconducting properties to efficiently absorb light, generate electron‐hole pairs, and separate them to perform the reduction and oxidation reaction of water splitting on the catalyst surface. This could be done by manipulating and optimizing the electronic and catalytic properties of catalysts by tuning bandgap, doping, and/or defect engineering [326], and constructing heterogeneous structures along with interfacial engineering [327]. Additionally, coupling with plasmonic materials may also be considered to further increase the light absorption and subsequently the catalytic activity. In terms of practical applications, the rational design of core‐shell architectures and double‐hollow yolk‐shell nanostructures could potentially promote the charge separation state at the heterojunctions toward highly efficient H_2_ production [328, 329, 330, 331]. In the case of electrocatalytic H_2_ production, electrons should be brought efficiently and effectively from the external circuit to the cathode surface and then directed to the active sites to undergo HER. Smooth electron transport along the cathode requires materials with good electrical conductivity and its anisotropic behavior [332]. The uniqueness of electronic properties of nanocatalysts such as TMD‐based materials enables the shift of semiconducting‐to‐metallic properties by their 2H‐to‐1T phase transition. In addition, different electronegativity of the catalyst components may also be used to optimize the intrinsic catalytic activity. Furthermore, phonon‐involved processes may be regulated to activate the reactant molecules. Besides that, the catalysts used should also facilitate the smooth mass transport of coming reactants and leaving products to ensure the continuity of HER over an intended period of time.

While recent progress in nanocatalyst design and characterization has provided a strong foundation, challenges remain in understanding fundamental HER mechanisms, optimizing scalable synthesis methods, and enhancing catalyst performance through advanced strategies such as interfacial engineering and computational modeling (e.g., DFT). They often overlook essential operational factors such as electrolyte interactions and applied bias, leading to discrepancies with experimental observations. Incorporating these elements into computational models is increasingly recognized as a key challenge and opportunity to improve the accuracy and relevance of theoretical prediction [333]. Future modeling efforts should therefore adopt advanced approaches that include operando electrochemical conditions to better guide material design and mechanistic understanding. Innovations in characterization techniques, like ultrafast pump‐probe spectroscopy and scanning electrochemical microscopy, are essential for linking catalyst structures to their activities at the nanoscale, which deepen our understanding of ultrafast HER phenomena, including identification of the rate‐limiting step and thereby inspire us to make impactful interventions in enhancing catalytic activity at the macroscale. Eventually, integrating photocatalytic and thermocatalytic systems and shifting toward the green hydrogen economy with the use of renewable feedstocks will be vital for aligning HER advancements with broader sustainability goals. Addressing these challenges can enable non‐noble metal nanocatalysts to match or exceed the performance of noble metals, facilitating transformative progress in energy and environmental technologies.

## Author Contributions

L. J. D and A.A.: Conceptualization, Visualization, Data curation, Investigation, Methodology, Formal analysis, Writing – original draft, Writing – review editing. G.H.A.W., T. H., M.A.K.S., F.S.H.S.: Conceptualization, Visualization, Writing – original draft, Writing – review editing. M. D. B.: Supervision, Resources, Writing – review and editing. K.S.N.: Supervision, Validation, Writing – editing.

## Funding

All authors acknowledge the Directorate of Research and Community Service 2025, grant under the Directorate General of Research and Development, Ministry of Higher Education, Science, and Technology, Republic of Indonesia, for the research grant through the basic research scheme, and the National Science Center, Poland, for the research grant OPUS‐24 no. 2022/47/B/ST5/01966.

## Conflicts of Interest

The authors declare that they have no known competing financial interests or personal relationships that could have appeared to influence the work reported in this paper.

## Data Availability

No new data were generated or analyzed during this study. This review formulates existing literature, and all data sources are cited within the text.
